# Rational inference strategies and the genesis of polarization and extremism

**DOI:** 10.1038/s41598-022-11389-0

**Published:** 2022-05-05

**Authors:** Peter D. Kvam, Abhay Alaukik, Callie E. Mims, Arina Martemyanova, Matthew Baldwin

**Affiliations:** 1 University of Florida, Gainesville, USA; 2 University of South Alabama, Mobile, USA

**Keywords:** Psychology, Human behaviour

## Abstract

Polarization and extremism are often viewed as the product of psychological biases or social influences, yet they still occur in the absence of any bias or irrational thinking. We show that individual decision-makers implementing optimal dynamic decision strategies will become polarized, forming extreme views relative to the true information in their environment by virtue of how they sample new information. Extreme evidence enables decision makers to stop considering new information, whereas weak or moderate evidence is unlikely to trigger a decision and is thus under-sampled. We show that this information polarization effect arises empirically across choice domains including politically-charged, affect-rich and affect-poor, and simple perceptual decisions. However, this effect can be disincentivized by asking participants to make a judgment about the difference between two options (estimation) rather than deciding. We experimentally test this intervention by manipulating participants’ inference goals (decision vs inference) in an information sampling task. We show that participants in the estimation condition collect more information, hold less extreme views, and are less polarized than those in the decision condition. Estimation goals therefore offer a theoretically-motivated intervention that could be used to alleviate polarization and extremism in situations where people traditionally intend to decide.

## Introduction

Attitudes toward crucial economic, environmental, and social policies are becoming increasingly polarized^1,2^. Extreme, and polarized views that are exacerbated by social and motivational forces can lead to civil unrest and violence^3^. Meanwhile, polarization and extremism have begun to manifest more frequently beyond the political sphere, extending to divisions and conflict among juries, boardrooms, religious organizations, nonpartisan commissions^4^, sports, and even consumer product brands (Apple vs Google, car brands, or the “console wars”)^5^. Even in entirely mundane decisions about perceptual stimuli without any political charge, opinions can become polarized^6^. We often have little choice in the matter: Individuals’ efforts and intentions to avoid forming extreme or polarized views can be rendered ineffective in the face of social and persuasive processes that exacerbate differences in opinion among groups^7^.

Polarization and extremism may not be so concerning if there were an easy solution to reversing their effects on cognition and behavior. Once it has set in, polarization distorts our view of the world and the way we consider new information, causing us to seek out confirming evidence^8–10^ both because confirming information is pleasurable and because it is felt to be more valuable than disconfirming information^11^. Managing the dissonance that arises from exposure to counter-attitudinal information leads to greater polarization, as decision makers with opposing attitudes begin to deal in extremes. Common ground is lost as information is selected not to inform but rather to reinforce negative attitudes toward one’s opponents^12,13^. In concert with these confirmation biases, people also strive to disconfirm information that is not in line with their existing beliefs^14^. In terms of reasoning, this can be perfectly rational: Bayesian reasoning prescribes that we should not ignore our prior beliefs when forming new ones, even when processing counter-attitudinal information^15^. However, prior beliefs or expectations can affect how people interpret new information, resulting in them taking more extreme positions or forming more extreme beliefs in light of information that conflicts with their existing beliefs^14,16–18^. People engage in motivated reasoning and belief revision driven by a desire to form beliefs that align with others^19^ and the utilities associated with holding and maintaining those beliefs^20^. This is exacerbated by the patterns of behavior on social networks, where communication and network dynamics lead to increases in polarization and the creation of echo chambers among users who agree with one another^21–23^. Attempts to reverse the process of polarization can even backfire^14,16,17^, as experimental findings have shown that groups of US Twitter users on opposite sides of the political spectrum became *more* polarized when exposed to opposing political views^24^.

Considering how insidious polarization can be once it has taken root, it is critical to understand and prevent the process of polarization at its source. In this paper, we seek to explain how individual-level cognitive mechanisms can create polarization in the first place, examining how people gather information as they consider new situations. Specifically, we evaluate how *inference goals* interact with decisions about when to continue or stop sampling information when choosing among options^25,26^, and how different goals can create or ameliorate polarization at the genesis of new opinions or views. Computational modeling of these choice processes has revealed that individual-level decision dynamics are related to political beliefs and ideological preferences across a range of topics like authoritarianism and dogmatism, social dominance, and openness to opposing views more generally^27^. Here, we go a step further to show that the very process of decision-making, even executed by a perfectly rational decision-maker, will create polarization and extreme views relative to the true information in the decision-maker’s environment. That is, rational individual decision-makers who try to make accurate and efficient choices will still become polarized and adopt extremist views. And although we build on work suggesting that rational goals can create polarization in social communication and group environments^28,29^, observing polarization in our findings would suggesting that rational individuals can form extreme and polarized views even in absence of communication or social pressure, in purely individual decisions, about relatively neutral and novel information ecologies.

If polarization is inevitable when making decisions, how can it be stopped? Fortunately, making a decision is not the only goal people can have when gathering new information. As opposed to determining which of two options is superior, one can evaluate or estimate their relative merit. Such a strategy incentivizes *precision* rather than *discrimination*, changing the conditions under which people stop sampling new information^26^. As a result, changing the incentives or directions for a task to favor estimation may prevent people from forming polarized views in the first place. This shifts the target of polarization interventions to one of prevention, rather than treatment: Estimation incentives should keep people from ever becoming polarized or forming extreme views (relative to the true information “out there” in the world) in the first place. In the following sections, we outline the reasoning behind this kind of intervention and how it avoids polarization and extremism caused by decision goals, briefly note the results of a re-analysis of seven decision-making studies illustrating the prevalence of polarization, and introduce a new study where we manipulate the goals of information collection to reduce the extremeness and polarization of views people form.

### The rational decision maker

Prior work often paints a picture of polarization as a phenomenon that arises from biases in information processing or belief updating^30^, motivation^8,9^, ingroup-outgroup processes^31,32^, and media algorithms and design^33^. Each of these explanations for polarization assumes that polarization emerges from bias or irrationality in the way decisions are made, while others explain how these beliefs can be propagated or exacerbated by rational reasoning based on already-polarized prior beliefs^14,16,17,27,34–37^. While such biases or priors certainly perpetuate divisions between people and exacerbate the problem of extremism, they do not entirely explain the genesis of polarization and how similar people can wind up on opposite sides of an innocuous issue at the outset. In this paper, we suggest that polarization can be created on entirely novel issues from basic judgment and decision making processes themselves. Moreover, we argue that these processes can act independently of social forces, such as motivation or group-processes, and can lead to polarization even in the absence of initial biases or social interaction. To support this claim, we prove mathematically that decision-makers will be biased toward extreme views when implementing optimal choice strategies aimed at maximizing decision quality and efficiency. This proof is provided in its completeness in the supplementary material, but we summarize it here.

**Figure 1:**
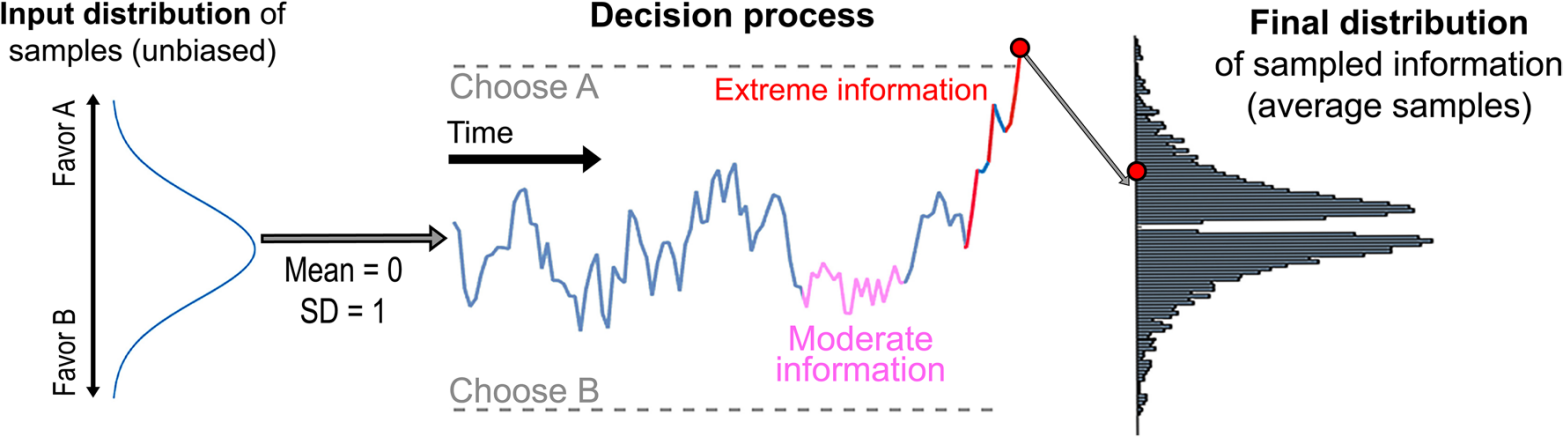
Illustration of how an unbiased distribution of information (left) becomes a polarized distribution (right) when fed through the decision process (middle). Because of an incentive to choose between options, moderate information—since it does not give credence to either options—gets discarded (middle).

When a decision maker is presented with a choice between two (or more) options, they must make a compromise between investing time on that decision and making a choice so that they can move on. Both time and belief accuracy bestow utility—the utility of making good (accurate) decisions is clear, but making a quick decision allows a decision-maker to proceed to the next choice, or move on to a task that they enjoy more than the choice. If a decision maker focuses purely on collecting information, they will never be able to stop and make a choice, whereas if they focus entirely on decision speed then they will have at-chance accuracy and make generally poor choices. Under the general framework of random walk/diffusion processes that are commonly used to describe and explain choice behavior in humans and other animals^38–40^, the optimal trade-off is achieved by setting a pre-defined *threshold* that corresponds to the level of accuracy that the decision maker desires, and then gathering information until that threshold is reached^41,42^. These models are based on the sequential probability ratio test^42,43^, where a decision maker tracks the balance of support between two options and uses this balance to determine when to make a choice. As they get information favoring option A, they shift up toward the “Choose A” response boundary, as shown in Fig. 1. Similarly, as they get information favoring option B, they shift down toward the “Choose B” response boundary. Once they cross one boundary or the other, the corresponding alternative is chosen (see the supplement for a full mathematical proof and description of simulations).

This strategy is specifically optimal for maximizing *reward rate*, or the amount of rewards that a decision-maker can accumulate per unit of time (seconds, minutes, days). There are slight modifications to the strategy, such as collapsing choice boundaries^44,45^, that are made to adapt it to different environments, but all reward rate optimality models share the elements of (a) tracking the balance of support, (b) accumulating the relative balance to a threshold, and then (c) deciding when the balance crosses a boundary. However, as we illustrate below, these three elements lead decision-makers to a distorted view of the evidence in their environment despite the decision process being optimal.

This unintended outcome occurs because a decision-maker’s goal is not to gather a representative sample of information, but to tip the balance of information far enough in one direction to conclude $A \succ B$ or $B \succ A$ (where $\succ$ denotes a preference order or belief). That is, choice tasks will bias information samples in the direction of the choice. On one hand, this is not so surprising, because we should expect people to have choice-consistent samples: Those who sample more of choice A information should choose option A. However, a deeper analysis reveals a surprising corollary to this expected pattern, namely, that the information sampled up to the point of choice is not representative of the true information in the world. Rather than mirroring the information in the environment (about choice A for instance), a decision-maker’s sampled information is more extreme^25,26^. In other words, the *goals* of the decision-makers influence the final sample of information that they collect, creating an unrepresentative sample of information despite no inherent biases.

One explanation for why this occurs is because moderate information is not conducive to achieving an agent’s decision goals (to stop and support A $\succ$ B, or B $\succ$ A), while extreme information makes it easy to stop and select one option over the other. These ideas are illustrated in Fig. 1: Moderate information (pink) cannot tip the balance of evidence sufficiently toward one option or another to trigger a choice, while extreme information (red) is very likely to do so. As a result, people who have made a decision using a balance-of-evidence stopping rule will have a greater number of extreme information samples than the underlying distribution.

In addition to polarization, optimal decision strategies can create subgroups of highly polarized individuals whose information samples are at the extreme ends of the spectrum. When a person gathers a piece of strong information in their first sample, there is the chance that this is the only piece of information they need to consider to make a choice. As a result, some individuals are likely to construct their views and make their decisions based on a minimal sample of particularly extreme information. In the simulations in the supplementary material, we examine factors that exacerbate the problem of these “extremists” who gather very little information and yet hold much more extreme views than other decision makers. In general, these extremist decision makers tend to be implementing optimal strategies that place greater subjective value on time over new information relative to other decision makers, reflected in lower thresholds that generate fast but not necessarily accurate responses^46,47^.

In the supplementary material, we provide a re-analysis of seven existing studies that allowed participants to make decisions by sampling information piece by piece over time. These studies show that the information participants consider is more extreme than the information naturally provided by their environment, lending credence to our prediction that decision-makers will become polarized. Furthermore, these studies show that the participants with the most extreme views are the same participants who sample the smallest amount of information, showing that extremism can arise even on simple perceptual and preferential choices. But the emergence of polarization and extremism is not inevitable. In the next section we describe one way that the decision process can be manipulated to incentivize the sampling of more representative information from the world.

### Interventions to reduce polarization

The seven re-analyzed studies illustrate that polarization and extremism both arise frequently around even mundane choices (numbers of dots, which posters/foods are best), and it may seem inevitable in all decisions. Fortunately, decision-making is not the only goal that people can pursue when making inferences. Strategies that are non-optimal for reward rate maximization, but satisfy other criteria, can be useful for reducing polarization among groups of people. For example, a strategy elicited by incentivizing estimation accuracy—where a person is asked to assess *how much* the information in their environment favors one option relative to another—can lead participants to gather a distribution of information that is representative of the true information in the environment.

**Figure 2:**
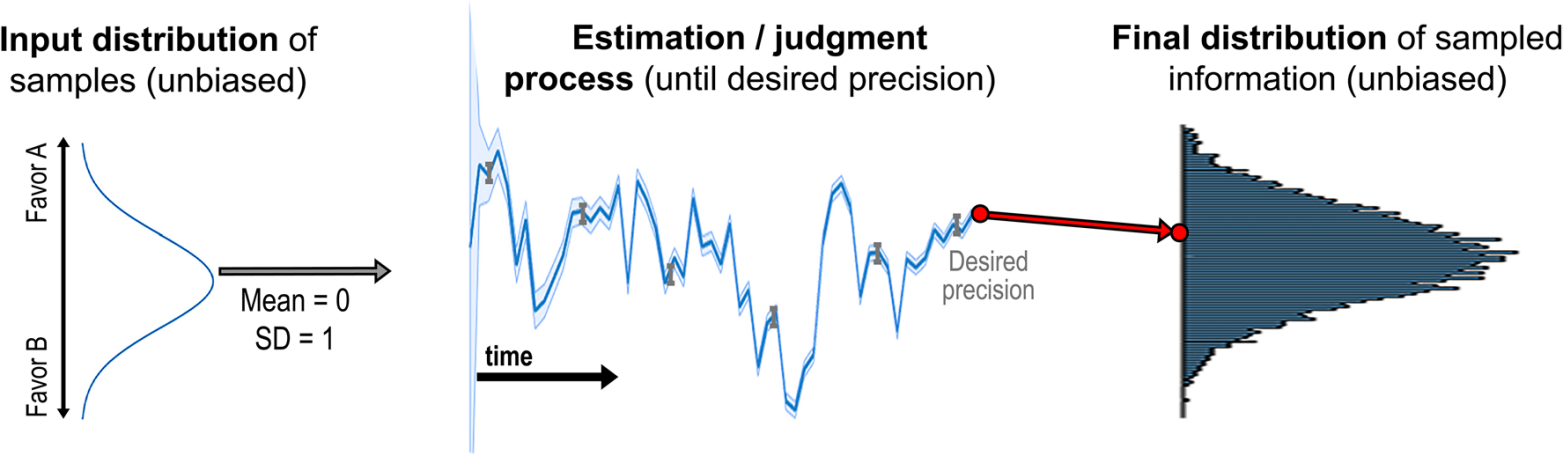
Illustration of a precision-based judgment process, where the goal is not to select one side over the other (as in Fig. 1) but to meet a criterion level of estimation precision.

Some support for the efficacy of an estimation goal in reducing the sampling issues in decision making is provided by Coenen and Gureckis^26^. They found that participants gathered largely representative samples while estimating relative strength of information, as opposed to a bimodal distribution of samples while choosing between two options. Model-based simulation results provide further evidence for its efficacy. As illustrated in Fig. 2, an optimal strategy for making an estimate (minimizing response time for a desired level of error) requires a participant (i.e., the judge) to continue sampling new information until a desired level of precision is achieved. This process involves collecting new information and shifting their estimate over time (blue line) until the error of their estimate of the mean (shaded blue area can correspond to standard error or posterior variance of the mean) falls below a criterion level. In this case, extreme information is counterproductive to making precise estimates because it fails to reduce the variance of a sample, and thus the posterior variance of the mean. Conversely, information that is close to the current posterior mean results in maximal reduction of the posterior variance of the mean estimate. As a result, the judge is incentivized to gather more information to form an estimate after gathering extreme information, and incentivized to stop after gathering moderate information.

Following this optimal strategy, the distribution of sampled information across a population of decision makers (right side of Fig. 2) will correspond closely to the input distribution (left side of Fig. 2). Posing the task as an estimation problem can therefore be used as an effective manipulation that reduces polarization and extremism by bringing the evidence sample closer to the true information distribution. In this paper, we test the effectiveness of this intervention across multiple types of inference problems, encompassing both simple perceptual problems and more politically or affectively charged situations. In the next section, we introduce an experiment that examines these possibilities and explores the individual differences that drive or exacerbate polarization and extremism in some people.

## Methods

All methods were carried out in accordance with relevant ethical guidelines and regulations. All experiments involving primary data were approved as exempt by the University of Florida Institutional Review Board (IRB202002176). These experiments were deemed to involve minimal risk to participants. Prior to completing the experiments, all participants were briefed on the study procedures and completed informed consent.

The experiments we present here were collected in two parts. An initial study collected data from 40 participants in each condition, separately for the choice and estimation conditions. We then collected data from a preregistered replication, randomly assigning an additional 110 participants to either the choice or estimation condition. However, the behavioral data from these two stages of data collection were nearly identical. We therefore present them altogether here for the sake of brevity. The data files from the first stage of data collection (Stage 1) and from the preregistered replication (All Data) are both provided on the Open Science Framework at osf.io/jsb52, so readers can view and analyze the two data sets separately as they wish. The preregistration is available at osf.io/qfb6w.

Our initial re-analysis showed that choice tasks, by their very structure, lead to polarized and extreme information sampling, even when applying rational and optimal sampling strategies. Moreover, our own simulations as well as work by previous authors^26^ suggest that estimation tasks could serve as a solution to this problem by reducing the extent to which people make decisions on the basis of extreme information. We further elucidate these processes in a new study that allows us to test whether polarization arises naturally in new environments with politically and affectively charged decisions. By comparing decision and estimation conditions, we evaluate whether estimation is a strategy that can alleviate polarization and extremism. The new study allows us to formalize our definitions of polarization and extremism (outlined below), and examine them as individual differences that can be related to relevant psychological traits, such as dogmatism, need for structure, and other demographic variables^27^. In the sections to follow, we detail each of these measures and examine their relationship to information sampling and individual-level metrics of polarization and extremism.

The structure of choice and estimation tasks were matched closely: In both conditions, corresponding scenarios were worded almost identically, the layout of the window was kept identical, and the sampling action (clicking on a button to collect information about Policy A vs. B) was kept the same as well. The key difference between the two conditions was in the way participants responded to each scenario. In the choice condition, they were asked to decide between two courses of action (“Select Policy A or Policy B”). In the estimation condition, they were asked to estimate the relative expected effect of implementing the two courses of action on a relevant criterion (rate how good is Policy A vs Policy B). This allowed us to directly compare patterns of information sampled in the choice and estimation conditions for every question that we asked.

### Task and items

In both conditions, participants were first instructed to imagine that they had encountered an alien civilization seeking their advice on several of their planet’s pressing issues. The use of an “alien world” cover story is designed to minimize the influence of biases that participants might bring to each scenario and to make it clear to participants that they are gathering information with the goal of forming beliefs about an entirely new issue. The alien worlds paradigm has been commonly used to examine processes related to the evaluative information ecology^48^, acquisition of morality^49^, language learning^50^, and reinforcement learning^51^.

Participants were informed that the aliens were considering two possibilities for each issue (i.e., “The aliens are building a new power plant that will generate energy from one of two materials—Material A and Material B”) and participants were tasked with helping the aliens decide by sampling information about each of the options. Participants had access to a button that would generate the relevant information (e.g., “Each unit of Material A produces *X* MORE/LESS units of energy than each unit of Material B”). Each time participants clicked the button, the random variable *X* was drawn from a normal distribution with mean 0, $X\sim N(0,SD)$. This meant that the true information that participants could sample was normally distributed and (on average) did not favor one option over the other. Participants had the freedom to sample as much or as little as they wished before responding.

**Figure 3:**
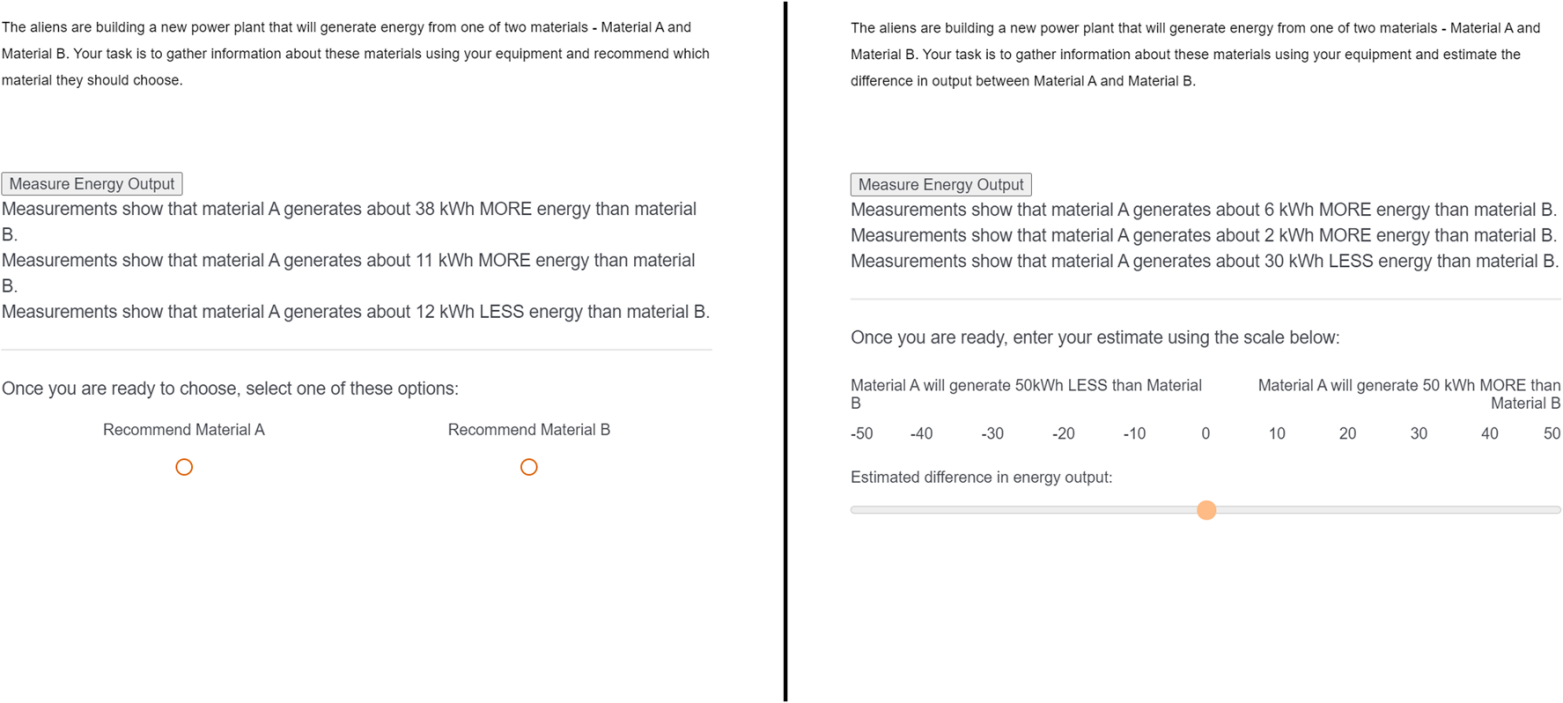
The scenarios in both tasks are worded very closely. The key difference lies in the task: after gathering information by clicking the button, participants either choose one of two options (left), or estimate how much better one option is than the other (right; the scale ranges from − 2.5 to + 2.5 SD of the true information distribution).

Crucially, participants in the choice condition were asked to choose the option which they thought was better (i.e., Material A or B), whereas participants in the estimation condition were asked to assess the degree to which one option was better than the other using a sliding scale (Fig. 3) that encouraged participants to view the scenario as distinct from a binary choice. The actual choice or estimate that participants made was largely unimportant to the analyses we present below—instead, our aim was to evaluate the relative degree of polarization and extremism between the choice and estimation conditions by comparing patterns of information search in each scenario.

Participants in both conditions experienced a total of 80 scenarios: 40 in which the button enabled them to sample information from the world (i.e. gathering information using their “equipment” or “tools”) and 40 in which the button allowed for sampling information from others (i.e. participants could consult with local alien experts to obtain relevant information). The scenarios were designed to incorporate a variety of issues that differed in terms of their subject matter as well as the degree to which they evoked affective involvement^52,53^: the 80 questions included 20 affect-poor, 16 affect-rich, 22 politically charged, 8 risky, 10 investment, and 4 attention-check scenarios. A complete list of the choice scenarios, along with the data and analyses presented here, is provided on the Open Science Framework at osf.io/jsb52. Representative examples of scenarios under each category are shown in Table 1.

**Table 1:** Representative examples of issues under each category of scenario.

Category	Representative example
Affect-rich	Pursue gene therapy or immunotherapy to save aliens from a deadly virus
Affect-poor	Mining for the mineral Xenon in Location A or B
Politically charged	Allowing immigrant aliens into the city or not
Risky	Settling in city A or B based on earthquake frequency
Investment	Investing in flood prevention infrastructure now or spend money later on flood response

### Participants

We recruited a total of 95 Prolific Academic workers for each condition for a total of 180 participants. This was composed of two stages of data collection: an initial sample of 40 participants in the choice and estimation conditions, assigned to conditions based on when they signed up to participate; and a preregistered replication study that randomly assigned 110 participants to either the choice or estimation condition. Our goal was to obtain data from approximately 85 participants in each condition, which is sufficient to identify relationships between individual-difference measures when they are present and to support the null hypothesis with a Bayes factor of 3 or more (moderate evidence) when there is no relationship between measures. A formal power analysis is provided in the supplement.

Ten participants from the choice condition and eleven from the estimation condition were removed because they failed attention checks or failed to respond at all on multiple trials. Hence, we retained responses from 85 individuals in the choice condition and 84 in the estimation condition, very close to our goal of 85 participants per condition. Participants in the choice condition were 54 women (30 men, 1 non-binary/other) with a mean age of 33.62 (*SD* = 11.63). For the estimation condition, participants were 45 women (37 men, 2 non-binary/other) with an average age of 36.59 (*SD* = 12.71). All participants indicated that they were from the United States and fluent English speakers. Participants were paid $10 per hour for their participation in the experiment.

Our sample size was intended to allow us 80% “power”—in this case, the ability to conclude in favor of the null using a Savage-Dickey Bayes factor analysis^54^ when there was no relationship between variables in a regression. Moreover, we collected responses to 80 trials for each participant, which results in approximately 13,500 data points in total to estimate the effects. Such a large set of data helps make our parameter estimates of both group-level and individual-level characteristics robust and reliable. Moreover, most of our analyses involve hierarchical (mixed effect) Bayesian models, which are typically stricter than classical statistics under uninformative priors^55^. In the results, we also provide 95% credible/highest density intervals [HDIs] for our point estimates, which indicate the 95% posterior most likely estimates of a particular parameter or statistic.

### Individual difference analyses

Similar to the re-analysis of the seven empirical studies in the previous section, we compared the observed distribution of information collected by participants against the expected distribution of an unbiased, representative sample that we would expect from a random sample of information. These distributions were compared in two main ways. First, we computed the difference between the distributions using an information-theoretic metric, the Kullback-Leibler divergence [KLD]. This quantifies the difference between the expected probability density of samples (a unimodal, normal distribution) and the observed density of samples. It is used as our measure of polarization, as it quantifies the degree to which the samples gathered by participants diverge from an expected unimodal distribution.

**Figure 4:**
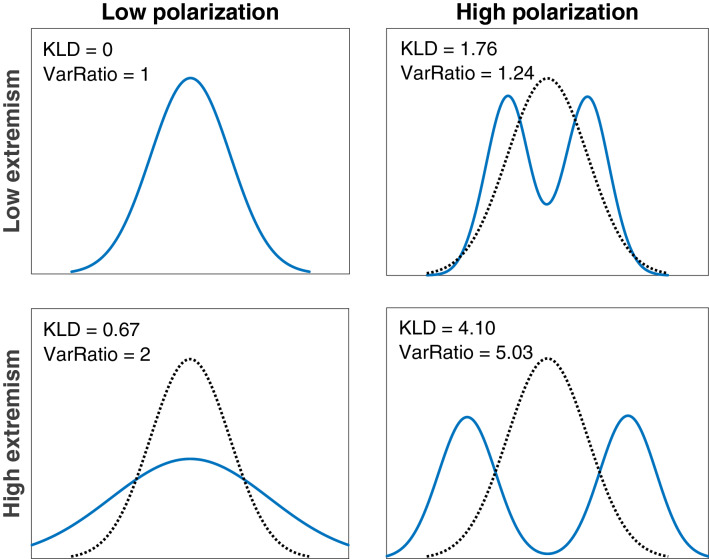
Diagram of the measures of polarization (low = left, high = right) and extremism (low = top, high = bottom), how different distributions of information might exemplify each of these properties, and how KLD and variance ratio (VarRatio) describe/quantify these properties. The dotted black line corresponds to the expected distribution, a normal distribution centered at zero, while the blue line corresponds to the observed distribution with high or low polarization/extremeness.

Second, we also quantify the extremeness of the sampled information by evaluating its variance, divided by the true variance of the pool of information participants are sampling from (i.e., the variance of a representative sample). This allows us to assess the degree to which participants’ sampling strategies led them to draw samples that are more (or less) extreme than the true information provided by their environment. Note that this measure of extremeness is often correlated with, but distinct from, our measure of polarization. As shown in Fig. 4, a distribution of information can be highly polarized/bimodal without high variance, and likewise it can have high variance without being bimodal or polarized.

Putting it all together, we formally define polarization and extremism in these studies as follows:

- *Polarization* Polarization is the extent of divergence between the distribution of the information collected and the true (expected) information distribution. We operationalize it as the Kullback-Liebler Divergence [KLD], which provides a measure of the overall difference between two probability distributions that we can use to compare observed and expected information distributions:
  1 $$\begin{aligned} KLD[p(y)||p(x)] = \int _{i}^{N} p(\mathbf{y} _i)\log _n \frac{p(\mathbf{y} _i)}{p(\mathbf{x} _i)} \end{aligned}$$
  where $p(\mathbf{y} _i)$ describes the probability density of a standard normal distribution and $p(\mathbf{x} _i)$ is a nonparametrically estimated probability density, generated using a Gaussian kernel density estimator with optimal bandwidth^56^, of sampled data. The indices *i* : *N* represent each of points on the probability distributions where their divergence is calculated.
- *Extremism* The ratio of the variance of the observed sample to the variance of a representative sample of information from that trial—termed *variance ratio* for the purposes of this study. If the overall variance ratio in the condition (i.e., condition-level) is above 1, the information sampled in the condition will be deemed extreme.

Both of these metrics can be applied at the level of a trial, participant, or entire condition: We simply evaluate the KLD or variance of a single trial relative to the expected distribution on that trial, all trials from a participant relative to the expected distribution for that participant, or all trials from all participants in a condition, respectively. In all cases, we compare the observed to expected distributions *conditional on how many samples were drawn*. This allows us to disentangle the number of samples drawn from the shape of the distribution of samples they drew. It also allows us to examine the number of samples as a separate outcome variable. The number of samples (the number of clicks on the “draw another sample” button in Fig. 3) served as a simple and direct measure of how much information people chose to gather before making their choice or estimate. We also measured the length of time participants spent on each trial in seconds, although this data was not analyzed.

For the extremism analysis/variance ratio, we can estimate uncertainty in the variance parameter simply by fitting it using Bayesian MCMC sampling methods. However, the KLD is a point estimate and thus does not convey uncertainty, making it hard to directly compare across conditions. To estimate a 95% HDI on the KLD for decision and estimation tasks, we instead simulated 10,000 artificial data sets with the same characteristics (number of trials, number of samples per trial) as the observed data. However, these artificial data sets were sampled from the true underlying distribution of information from the stimulus, i.e., they did not use the same stopping rules as participants. In other words, they did not have any sampling biases that our participants might have had. This yielded 10,000 “samples” from the true distribution against which we could compare the observed results. We computed the mean difference between observed and expected KLDs by subtracting the mean of the KLDs across all 10,000 artificial data sets from the KLD of the observed data set. Then, we constructed the 95% HDI on the difference between the observed and expected distribution by computing the 95% highest density interval of differences between observed KLD and each of the 10,000 artificial data set KLDs.

At the conclusion of the study, participants completed self-report measures of dogmatism^57^ and personal need for structure^58^ and reported their political ideology and basic demographic information. We included dogmatism in our analysis because previous work has connected it to reduced information sampling under uncertainty^59^. Need for structure was included because need for closure, of which the need for structure appears to be a more psychometrically desirable variant^58^, has previously been connected to the threshold parameter in dynamic decision models^60^. We report the relationships between all of these individual differences—extremism/variance ratio, polarization/KLD, information sampling/number of clicks, dogmatism, and need for structure—in the results.

## Results

All the analyses were conducted in R^61^, JASP^62^, and MATLAB. Data were analyzed with Bayesian estimation of the posterior using uninformed priors and hierarchical/mixed models, where appropriate. We implemented MCMC sampling in JAGS^63^ to generate all statistics of interest (such as variance ratios and correlations). We report mean estimates and their corresponding 95% HDI^55^. Code for these analyses are provided on the Open Science Framework at (osf.io/jsb52), and we expand on their descriptions in the supplementary materials.

### Polarization and extremism

**Figure 5:**
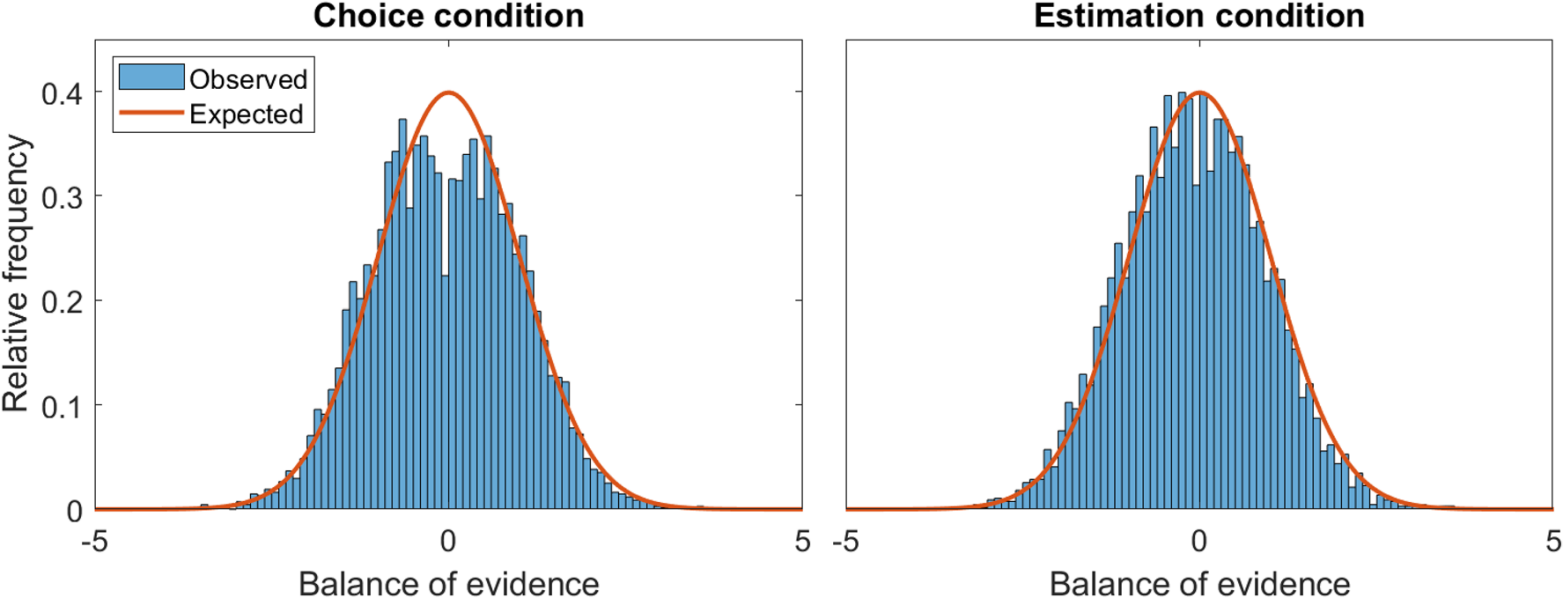
Distribution of information collected by participants (blue histogram) in the choice (left) and estimation (right) conditions, compared against the expected distribution that would result from unbiased, representative sampling (orange line).

We first compared the distributions of information collected by participants against the distributions we would expect from a random, representative sample across trials. These distributions can be seen in Fig. 5. The blue histograms represent the distributions of information sampled by participants, while the orange line represents the true (standard normal) distribution from which the samples were drawn. The difference is immediately striking—as in the re-analyzed studies and the model prediction shown in Fig. 1, there is a “dip” in the middle of the distribution of information collected by participants in the choice condition. This bimodal distribution of information shows that people are under-representing moderate information when sampling.

By comparison, the estimation condition shows little to no substantial deviation from the expected distribution, indicating that the estimation intervention appears to evoke more representative sampling than the choice condition. That is, moderate information was considered equally in making estimations, resulting in a typical bell-shaped distribution of information that reflected the true underlying information in the environment.

Our quantitative measure of polarization (KLD) also aligned with these visual observations: the expected KLD in the decision condition was approximately 0.12, but the observed KLD was 1.00 ($M_{diff}$ = 0.88, 95% HDI = [0.80, 0.95]). The expected KLD in the estimation condition was approximately 0.12, whereas the observed KLD was 0.52 ($M_{diff}$ = 0.40, 95% HDI = [0.32, 0.47]). Thus, the deviation from the true distribution—and thus the amount of polarization, as defined by KLD—was more than twice as large in the decision condition compared to the estimation condition. KLD scores in the estimation condition were on average .26 standard deviations lower (95% HDI = $[-0.30, -0.23]$ than KLD scores in the choice condition, corresponding to a medium-sized effect^64^. A model comparison revealed that the regression yielded extremely strong support for inclusion of condition as a factor predicting KLD ($\log (BF)=358.30$) (Note that we report natural log Bayes factors here and elsewhere in the text, as often the Bayes factors on raw odds scales are approach infinite values. A Bayes factor of 30 on a raw scale ($+3.40$ on a natural log scale) indicates extremely strong support for an effect/inclusion of a model parameter, while a Bayes factor of 1/30 on a raw scale ($-3.40$ on a natural log scale) indicates strong evidence against an effect/inclusion of a model parameter^65,66^

Turning to extremism metrics, we found the condition-level variance ratio for the choice condition to be $M= 1.18$ (95% HDI = [1.11, 1.27]), where a value of 1.00 indicates that the observed sample is equally as extreme as a representative sample. Conversely, in the estimation condition, the variance ratio was centered right around the expected variance, $M = 1.01$ (95% HDI = [0.96, 1.09]). These HDIs are entirely non-overlapping, meaning that we can be very confident that the variance in the choice condition is higher than the variance in the estimation condition, signaling that the estimation task reduced the extremeness of information participants gathered.

We also evaluated patterns of extremeness by calculating correlations between the variance of samples people collected with the total number of times they chose to sample information (i.e., number of clicks). The extremeness of information participants collected was credibly correlated with the number of times people sampled information in each trial, both in the choice condition (*r* = – 0.44; *HDI* = $[-0.47, -0.41]$; *ln*(*BF*) = 296.16), as well as in the estimation condition (*r* = – 0.48; *HDI* = $[-0.51, -0.46]$; *ln*(*BF*) = 397.94). That is to say, the more people sampled extreme information, the fewer times they clicked to receive new information *within both conditions*. The amount (or lack of) of information collected is therefore predictive of extremism above and beyond choice-based polarization, suggesting that manipulations increasing information sampling are likely to be effective in both choice and estimation tasks.

### Information search across conditions

**Figure 6:**
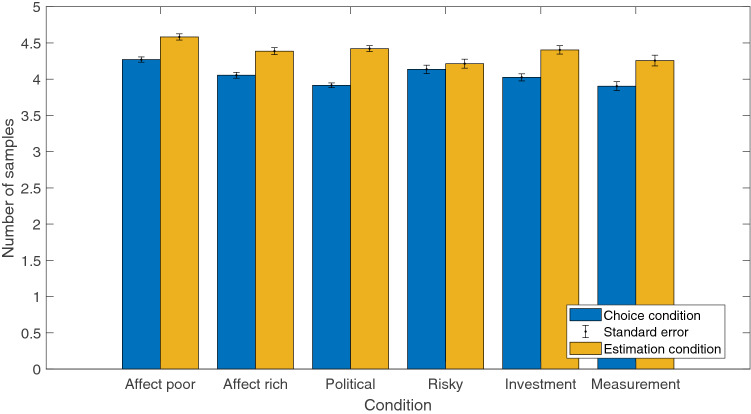
Mean number of samples drawn (error bars indicate standard error) for each question type in the choice (blue) and estimation (yellow) condition.

We hypothesized that one of the reasons for extremism and polarization is that some participants tend to gather less information than others (lower thresholds), and less information typically leads to more volatile/extreme samples of evidence. It is possible that the choice/estimation manipulation, or the question types, led participants to seek out more or less information. This can be examined by looking at the number of pieces of information that participants collected in each condition, quantified as the number of times they clicked on the button to sample more information before making their choice or estimate. A comparison between the different question types, as well as between choice and estimation conditions, on the number of pieces of information sampled is shown in Fig. 6.

To formally quantify whether sampling differed between choice and estimation conditions, we examined the number of samples that participants gathered in each condition. We ran a Bayesian Linear Regression in JASP^62^ with default/standard priors^67,68^, which provided strong evidence that condition should be included as a predictor of the number of clicks ($\ln (BF) = 13.12$). In the estimation condition, number of clicks increased on average by 0.32 clicks relative to the choice condition (95% HDI = [0.21, 0.44]). Therefore, part of the way that the estimation condition reduces extremism is by encouraging participants to seek out more information, resulting in more informed judgments.

Similarly, a greater number of clicks led to less polarization, reducing the KLD by an average of 0.06 standard deviations per click in the choice condition (95% HDI = $[-0.07, -0.05]$; $ln(BF) = 125.29$) and by an average of 0.09 standard deviations per click in the estimation condition (95% HDI = $[-0.09, -0.08]$, $ln(BF) = 284.67$). Gathering more information is a clear-cut strategy for reducing both polarization and extremism, which is why the increase in information search in the estimation condition was so effective at reducing both effects.

To examine whether the type of inference domain affected sampling behavior, we tested whether condition-level manipulations impacted information sampling by looking at the effect of question type (affect poor, affect rich, etc) on number of clicks. We ran a Bayesian ANOVA in JASP^62,69^ which indicated that question type had no effect on number of clicks ($BF(H_0 > H_1)$ = 270.66, indicating strong support for the null hypothesis). There were also no substantial differences on question type in predicting individual-level KLDs ($BF(H_0 > H_1)$ = 1237.94, indicating strong support for the null hypothesis) so we do not examine question type further.

### Individual differences

In addition to the differences between conditions, we hypothesized that information search and polarization/extremism should be predicted by personality factors like dogmatism (DOG) and personal need for structure (PNS), which is comprised of the desire for structure (DFS) and reaction to lack of structure (RLS). As summarized in Table 2, the model predicting information search indicated a negative effect of dogmatism, positive effect of the personal need for structure—desire for structure subscale, and negative effect of the reaction to lack of structure subscale. This aligns with recent findings showing that dogmatism is related to decreased information search under uncertainty^27,59^. Interestingly, the sub-scales of Personal Need for Structure predicted information search in opposite directions. It appears that a desire for structure leads participants to search for less information, perhaps because making a quick decision satisfies the desire to resolve uncertainty and reach a conclusion, while the discomfort associated with having ill-informed views based on little evidence (reaction to lack of structure) drives people to sample more.

**Table 2:** Estimates of the effects of Dogmatism, Personal Need for Structure Reaction to Lack of Structure subscale (PNS-RLS), and Personal Need for Structure Desire for Structure subscale (PNS-DFS) on polarization (K-L divergence, bottom) and information search (number of clicks on the sampling button on each trial, top).

Term	Estimate	SE	95% HDI
**Information sampling**			
Intercept	4.14	0.23	[3.70, 4.60]
Dogmatism	− 0.01	0.00	[− 0.01, − 0.00]
PNS-DFS	− 0.14	0.01	[− 0.16, − 0.12]
PNS-RLS	0.23	0.01	[0.20, 0.26]
**Polarization**			
Intercept	47.22	2.90	[41.43, 52.99]
Dogmatism	− 0.09	0.05	[− 0.18, − 0.00]
PNS-DFS	0.55	0.26	[0.04, 1.08]
PNS-RLS	− 0.70	0.36	[− 1.42, − 0.01]

These individual differences also predicted individual-level polarization. This was tested by again using a Bayesian linear regression to assess the credibility of individual differences as predictors of participant-level (standardized) KLD. As with information search, the regression suggested that all three measures predicted KLD: dogmatism led to decreased polarization, desire for structure led to greater polarization , and reaction to lack of structure led to less polarization.

The KLD results are particularly noteworthy because dogmatism predicts behavior in the opposite direction than we expected: Greater dogmatism predicted less polarization in resulting sampling behavior (lower KLD), while the PNS scales align with the information search results. So while dogmatism results in reduced information search, this reduction in information sampling does not translate directly to less representative views. One possibility is that highly dogmatic individuals hit a “sampling floor” and sampled only one piece of information before deciding. This would result in a low KLD, because lone samples will not favor extreme information more than moderate information and will, in fact, constitute representative simple random samples. By setting very low thresholds, dogmatic individuals therefore shift back toward a representative sample. As a result, they are ill-informed but no more polarized than a random sample of information from the environment would expect.

## Discussion

Our studies illustrate that regardless of the domain—spanning perceptual choices about dots or colors, preferential choices about food or posters (as revealed in the reanalysis of seven existing studies), neutral social policies, or politically and affectively charged decisions (as revealed in the empirical studies reported here)—the process of decision making generates polarization and extremism during the information sampling process. These were scenarios where there was no social interaction among participants, and where there should be little motivation to engage in biased information processing. While a tremendous volume of work has gone into understanding motivated reasoning^70^, communication dynamics and social networks^21,28,29^, and the social forces that shape and exacerbate polarization^31,32,71,72^, it has focused less on individual-level information search mechanisms that might create polarization before beliefs impacted by social influence. Our work suggests that the root causes of polarization may be even deeper than previously thought, arising from completely rational, individual-level decision strategies.

As we show, polarized and extreme individuals may be those who are merely reacting rationally to choice incentives, and are not necessarily intentionally malicious actors. Based on the model simulations, we should expect that extremists will show up most frequently when there is time pressure to make choices and when decision makers begin the choice process with a biased position. Nevertheless, rational decision making processes—resulting from dual pressures on decision quality and time—will create polarized groups of decision makers and generate a subset of uninformed individuals who hold extreme views.

A major concern regarding these extremists is the degree of influence that they can carry in cultural discourse and the formation of public opinion. Decision makers with small, extreme samples of information are still confident in their views^73,74^, possibly due to a belief in the “law of small numbers” where people think even very small samples will possess the same statistical properties as large samples^75^. This suggests that extremists will have strong convictions and be willing to spread their views, despite the information they have gathered possessing relatively poor reliability. This is exacerbated by several other established phenomena. First, those with the most extreme positions carry a disproportionately greater sway over group discourse because their outlying beliefs are more robust to change and can thus “pull” other people toward them^76^. Second, extremists will be the first to share the information they have—by virtue of taking less time to make decisions, they are free to share their views before those who have taken more time and carefully constructed their beliefs with large samples of information. Extremists will therefore be the first to influence their peers via word of mouth and social networks^77^, provide reviews of new products that influence many subsequent buyer decisions^78^, and spread hateful views that carry quickly through social networks^79^. Social media algorithms base recommendations on early posters, meaning that their “hot takes” will carry greater weight in determining subsequent users’ views and activity as well^77,80^.

As a result, we arrive at a scenario where the least-informed (fewest samples of information), most dogmatic, and most impatient (lowest-threshold) individuals in a population will have the greatest influence on public opinion. The high profile of extremists can explain why perceived political polarization is exaggerated–people tend to think that the political left and right in the US are far more polarized than they are^81^.

### Alleviating polarization and extremism

The estimation incentives we examined in the new study suggest that polarization and extremism can be reduced at the outset by changing the optimal sampling strategy. Simply asking people to give their best estimate of the difference between options, as opposed to deciding between them, drives a more natural information sampling approach that reduces polarization and extremism while promoting greater information search. Successful implementation of this type of intervention provides avenues for reducing the divides between groups of people, and potentially can be brought to bear in designing simple interventions or judgment architectures to reduce these social problems^82^.

In the political sphere, our results suggest that binary or multiple-choice voting—requiring voters to decide in favor of only Candidate A or Candidate B (or C, D, E, etc)—may be partly responsible for the current state of political polarization. Fortunately, the estimation results suggest a remedy for this particular component of polarization. Specifically, it may be possible to alleviate political polarization through approaches like cardinal voting^83^, which incentivizes precise ratings (estimation) as opposed to forcing voters to select one option over another. Based on our estimation results, there is reason to hope that this could encourage greater information search among voters as well as more representative sampling. Certainly future work ought to evaluate the practicality and efficacy of different voting interventions like these that could reduce polarization on social issues.

An intriguing possibility that is raised by this work is that many types of information search may yield polarized samples. This may apply beyond literal information search—as we presented here—into domains like information search from memory, which is thought to leverage analogous mechanisms to external information search^38,84^. If search through memory is terminated using the same rules as diffusion or random walk models, then a person’s judgment goals at the time of measurement (when they are asked to make a choice or estimate) may determine what information they consider. Similarly, it may be possible to override samples of biased information that have been collected by changing the communication process. Asking a polarized individual to share information to someone who’s goal it is to *estimate* may incentivize sharing of representative information from memory. It may therefore be possible to alleviate polarization and extremism by manipulating inference and communication goals in more applied situations like the ballot box, boardroom, or social media feed. To the extent that the interventions we design here are useful for manipulating information search in these domains, they are promising for reducing polarization and extremism.

### Conclusions

In a decision scenario that incentivizes a trade-off between time and decision quality, rational decision makers will sample more polarized and extreme information than their environment naturally provides. However, we have also identified a potential solution that is as simple as changing decision goals: Polarization and extremism disappear when people are asked to estimate some relative quantity or preferability instead of choosing between options. This approach to making inferences encourages information search while simultaneously reducing the biases inherent to decision-making. Mathematical models, simulations, re-analysis of data from seven empirical studies, as well as results from a new experiment point to the advantages of estimation tasks over choice/decision tasks if one wants to reduce polarization and extremism. Future work will determine how widely and effectively this type of intervention can be implemented to reduce polarization and extremism outside the laboratory, but we hope that this work sheds light on one route to responsibly construct inference environments to create more common ground that brings people together instead of driving them apart.

## Supplementary Information

Supplementary Information.

## References

[1] Baldassarri D, Gelman A (2008). Partisans without constraint: Political polarization and trends in American public opinion. Am. J. Sociol..

[2] Wilson AE, Parker V, Feinberg M (2020). Polarization in the contemporary political and media landscape. Curr. Opin. Behav. Sci..

[3] Berger JM (2018). Extremism.

[4] Sunstein, C. R. The law of group polarization. *University of Chicago Law School, John M. Olin Law & Economics Working Paper* (1999).

[5] Shi, Y., Mast, K., Weber, I., Kellum, A. & Macy, M. Cultural fault lines and political polarization. In *Proceedings of the 2017 ACM on Web Science Conference*, 213–217 (2017).

[6] Baron RS, Roper G (1976). Reaffirmation of social comparison views of choice shifts: Averaging and extremity effects in an autokinetic situation. J. Pers. Soc. Psychol..

[7] Isenberg DJ (1986). Group polarization: A critical review and meta-analysis. J. Pers. Soc. Psychol..

[8] Festinger L (1957). A Theory of Cognitive Dissonance.

[9] Nickerson RS (1998). Confirmation bias: A ubiquitous phenomenon in many guises. Rev. Gen. Psychol..

[10] Westerwick A, Johnson BK, Knobloch-Westerwick S (2017). Confirmation biases in selective exposure to political online information: Source bias vs. content bias. Commun. Monogr..

[11] Lord CG, Ross L, Lepper MR (1979). Biased assimilation and attitude polarization: The effects of prior theories on subsequently considered evidence. J. Pers. Soc. Psychol..

[12] Iyengar S, Sood G, Lelkes Y (2012). Affect, not ideology: A social identity perspective on polarization. Public Opin. Q..

[13] Kuhn D, Lao J (1996). Effects of evidence on attitudes: Is polarization the norm?. Psychol. Sci..

[14] Gershman SJ (2019). How to never be wrong. Psychon. Bull. Rev..

[15] Taber CS, Cann D, Kucsova S (2009). The motivated processing of political arguments. Polit. Behav..

[16] Cook J, Lewandowsky S (2016). Rational irrationality: Modeling climate change belief polarization using Bayesian networks. Topics Cogn. Sci..

[17] Dixit AK, Weibull JW (2007). Political polarization. Proc. Natl. Acad. Sci..

[18] Botvinik-Nezer, R., Jones, M. & Wager, T. Fraud beliefs following the 2020 us presidential election: A belief systems analysis. *PsyArXiv* (2021). 10.1038/s41562-023-01570-4 37037989

[19] Wheeler NE (2020). Ideology and predictive processing: Coordination, bias, and polarization in socially constrained error minimization. Curr. Opin. Behav. Sci..

[20] Zmigrod, L. Mental computations of ideological choice and conviction: The utility of integrating psycho-economics and bayesian models of belief. *Psychol. Inquiry* (in press).

[21] Kashima Y, Perfors A, Ferdinand V, Pattenden E (2021). Ideology, communication and polarization. Philos. Trans. R. Soc. B.

[22] Brady WJ, Wills JA, Jost JT, Tucker JA, Van Bavel JJ (2017). Emotion shapes the diffusion of moralized content in social networks. Proc. Natl. Acad. Sci..

[23] Crockett MJ (2017). Moral outrage in the digital age. Nat. Hum. Behav..

[24] Bail CA (2018). Exposure to opposing views on social media can increase political polarization. Proc. Natl. Acad. Sci..

[25] Coenen A, Gureckis T, Papafragou A, Grodner D, Mirman D, Trueswell J (2016). The distorting effect of deciding to stop sampling. Proceedings of the 37th Annual Meeting of the Cognitive Science Society.

[26] Coenen, A. & Gureckis, T. The distorting effects of deciding to stop sampling information. *PsyArXiv* (2021).

[27] Zmigrod L, Eisenberg IW, Bissett PG, Robbins TW, Poldrack RA (2021). The cognitive and perceptual correlates of ideological attitudes: A data-driven approach. Philos. Trans. R. Soc. B.

[28] Burnstein E, Vinokur A (1973). Testing two classes of theories about group induced shifts in individual choice. J. Exp. Soc. Psychol..

[29] Burnstein E, Vinokur A (1977). Persuasive argumentation and social comparison as determinants of attitude polarization. J. Exp. Soc. Psychol..

[30] DeKay ML (2015). Predecisional information distortion and the self-fulfilling prophecy of early preferences in choice. Curr. Dir. Psychol. Sci..

[31] Hogg MA, Turner JC (1985). Interpersonal attraction, social identification and psychological group formation. Eur. J. Soc. Psychol..

[32] Suhay E (2015). Explaining group influence: The role of identity and emotion in political conformity and polarization. Polit. Behav..

[33] Benkler Y, Faris R, Roberts H (2018). Network propaganda: Manipulation, disinformation, and radicalization in American politics.

[34] Olsson, E. J. A Bayesian simulation model of group deliberation and polarization. In *Bayesian Argumentation*, 113–133 (Springer, 2013).

[35] Jern A, Chang K-MK, Kemp C (2014). Belief polarization is not always irrational. Psychol. Rev..

[36] Pallavicini J, Hallsson B, Kappel K (2021). Polarization in groups of Bayesian agents. Synthese.

[37] Singer DJ (2019). Rational social and political polarization. Philos. Stud..

[38] Ratcliff R, Smith PL, Brown SD, McKoon G (2016). Diffusion decision model: Current issues and history. Trends Cogn. Sci..

[39] Busemeyer JR, Gluth S, Rieskamp J, Turner BM (2019). Cognitive and neural bases of multi-attribute, multi-alternative, value-based decisions. Trends Cogn. Sci..

[40] Brunton BW, Botvinick MM, Brody CD (2013). Rats and humans can optimally accumulate evidence for decision-making. Science.

[41] Bogacz R, Brown E, Moehlis J, Holmes P, Cohen JD (2006). The physics of optimal decision making: A formal analysis of models of performance in two-alternative forced-choice tasks. Psychol. Rev..

[42] Edwards W (1965). Optimal strategies for seeking information: Models for statistics, choice reaction times, and human information processing. J. Math. Psychol..

[43] Wald A, Wolfowitz J (1949). Bayes solutions of sequential decision problems. Proc. Natl. Acad. Sci. USA.

[44] Tajima S, Drugowitsch J, Pouget A (2016). Optimal policy for value-based decision-making. Nat. Commun..

[45] Boehm U, van Maanen L, Evans NJ, Brown SD, Wagenmakers E-J (2020). A theoretical analysis of the reward rate optimality of collapsing decision criteria. Attent. Percept. Psychophys..

[46] Heitz RP, Schall JD (2012). Neural mechanisms of speed-accuracy tradeoff. Neuron.

[47] Wickelgren WA (1977). Speed-accuracy tradeoff and information processing dynamics. Acta Psychol..

[48] Alves H, Koch A, Unkelbach C (2018). A cognitive-ecological explanation of intergroup biases. Psychol. Sci..

[49] Rottman J, Kelemen D (2012). Aliens behaving badly: Children’s acquisition of novel purity-based morals. Cognition.

[50] Gupta P (2004). Space aliens and nonwords: Stimuli for investigating the learning of novel word-meaning pairs. Behav. Res. Methods Instrum. Comput..

[51] Decker JH, Otto AR, Daw ND, Hartley CA (2016). From creatures of habit to goal-directed learners: Tracking the developmental emergence of model-based reinforcement learning. Psychol. Sci..

[52] Zajonc RB, Markus H (1982). Affective and cognitive factors in preferences. J. Consumer Res..

[53] Pachur T, Hertwig R, Wolkewitz R (2014). The affect gap in risky choice: Affect-rich outcomes attenuate attention to probability information. Decision.

[54] Wagenmakers EJ, Lodewyckx T, Kuriyal H, Grasman R (2010). Bayesian hypothesis testing for psychologists: A tutorial on the Savage-Dickey method. Cogn. Psychol..

[55] Kruschke JK (2014). Doing Bayesian Data Analysis: A Tutorial with R, JAGS, and STAN.

[56] Bowman AW, Azzalini A (1997). Applied Smoothing Techniques for Data Analysis: The Kernel Approach with S-Plus Illustrations.

[57] Altemeyer B (2002). Dogmatic behavior among students: Testing a new measure of dogmatism. J. Soc. Psychol..

[58] Neuberg SL, Newsom JT (1993). Personal need for structure: Individual differences in the desire for simpler structure. J. Pers. Soc. Psychol..

[59] Schulz L, Rollwage M, Dolan RJ, Fleming SM (2020). Dogmatism manifests in lowered information search under uncertainty. Proc. Natl. Acad. Sci..

[60] Evans NJ, Rae B, Bushmakin M, Rubin M, Brown SD (2017). Need for closure is associated with urgency in perceptual decision-making. Mem. Cogn..

[61] Team, R. C. R: A language and environment for statistical computing (2013).

[62] JASP Team. JASP (Version 0.14.1)[Computer software] (2020).

[63] Plummer, M. JAGS: A program for analysis of Bayesian graphical models using Gibbs sampling. In *Proceedings of the 3rd International Workshop on Distributed Statistical Computing*, Vol. 124, 10 (2003).

[64] Cohen J (2013). Statistical Power Analysis for the Behavioral Sciences.

[65] Jeffreys H (1961). The Theory of Probability.

[66] Raftery AE (1995). Bayesian model selection in social research. Sociol. Methodol..

[67] Clyde MA, Ghosh J, Littman ML (2011). Bayesian adaptive sampling for variable selection and model averaging. J. Comput. Graph. Stat..

[68] Consonni G, Fouskakis D, Liseo B, Ntzoufras I (2018). Prior distributions for objective Bayesian analysis. Bayesian Anal..

[69] Rouder JN, Morey RD, Speckman PL, Province JM (2012). Default Bayes factors for ANOVA designs. J. Math. Psychol..

[70] Rigoli F (2021). Masters of suspicion: A Bayesian decision model of motivated political reasoning. J. Theory Soc. Behav..

[71] Tajfel HE (1978). Differentiation Between Social Groups: Studies in the Social Psychology of Intergroup Relations.

[72] Tajfel, H. E. & Turner, J. C. An integrative theory of intergroup conflict. In (M. A. Hogg & D. Abrams Eds.) *Intergroup relations: Essential readings*. 94–109 (2001).

[73] Kvam PD, Pleskac TJ (2016). Strength and weight: The determinants of choice and confidence. Cognition.

[74] Griffin D, Tversky A (1992). The weighing of evidence and the determinants of confidence. Cogn. Psychol..

[75] Tversky A, Kahneman D (1971). Belief in the law of small numbers. Psychol. Bull..

[76] Navarro DJ, Perfors A, Kary A, Brown SD, Donkin C (2018). When extremists win: Cultural transmission via iterated learning when populations are heterogeneous. Cogn. Sci..

[77] Lynn T, Muzellec L, Caemmerer B, Turley D (2017). Social network sites: Early adopters’ personality and influence. J. Prod. Brand Manag..

[78] Stephen AT, Lehmann DR (2016). How word-of-mouth transmission encouragement affects consumers’ transmission decisions, receiver selection, and diffusion speed. Int. J. Res. Market..

[79] Mathew, B., Dutt, R., Goyal, P. & Mukherjee, A. Spread of hate speech in online social media. In *Proceedings of the 10th ACM Conference on Web Science*, 173–182 (2019).

[80] Bastide, P. R., Deluca, L. S. & Do, L. M. User recommendations in a social media network (2018). US Patent 9,871,758.

[81] Westfall J, Van Boven L, Chambers JR, Judd CM (2015). Perceiving political polarization in the United States: Party identity strength and attitude extremity exacerbate the perceived partisan divide. Perspect. Psychol. Sci..

[82] Thaler RH, Sunstein CR (2009). Nudge: Improving Decisions About Health, Wealth, and Happiness.

[83] Baujard A, Gavrel F, Igersheim H, Laslier J-F, Lebon I (2018). How voters use grade scales in evaluative voting. Eur. J. Polit. Econ..

[84] Shiffrin, R. M. Memory search. *Models of Human Memory* 375–447 (1970).

