# Supplementary material for: Rational inference strategies and the genesis of polarization and extremism
Source: Sci Rep. 2022 May 5;12:7344. doi: 10.1038/s41598-022-11389-0 (PMC9072310; doi:10.1038/s41598-022-11389-0)
Supplement: Supplementary file 1 — Supplementary Information. [file 41598_2022_11389_MOESM1_ESM.pdf]

Supplementary materials for “Rational inference strategies and the genesis of polarization and extremism”

Peter D. Kvam[1,*], Abhay Alaukik[1], Callie E. Mims[2], Arina Martemyanova[1], and Matthew Baldwin[1]

[1]University of Florida
[2]University of Southern Alabama
[*]Corresponding author: pkvam@ufl.edu

ABSTRACT

In this document, we provide a proof giving the expected distribution of information among decision makers and examine simulations based on manipulations of the decision making process. We apply our analyses of polarization and extremism to seven existing studies, showing that choice creates a sampling bias even in simple perceptual decisions. We additionally show that our results will hold for multi-alternative choice, such that polarization and extremism are not confined strictly to the domain of binary choice, and examine alternative model-based solutions to alleviating polarization and extremism.

Proof of rational polarization & extremism

A rational choice has to maximize expected utility in terms of both the goodness of a decision (accuracy, expected outcomes) as well as the time it takes to make a decision. The subjective utility that a decision maker experiences will balance both factors, and so a rational decision maker must identify the balance between desired accuracy and decision time that will yield the highest expected utility[1,2]. To do so, a decision maker must compromise between spending more time on a choice and considering more information (lower utility from time, greater utility from accuracy) or spending less time and considering less information (greater utility from time, lower utility from accuracy). A decision maker should stop considering new information and make their choice when the expected benefit of considering one more piece of information (marginal improvement in accuracy) is outweighed by the loss of utility that accompanies spending the time required to consider that piece of information (marginal cost of time). Accuracy, quantified in terms of the odds of making a correct decision, is log-linearly related to the balance of support between options[3]. If each new piece of information can be expected to shift the balance of support toward one option (+1) or away from it toward another (-1), the marginal gain in accuracy (probability of correct) will decrease as the balance of support tips further and further toward one option. [1] A rational decision maker should therefore stop and make their choice when this marginal expected improvement in accuracy dips below the utility of the time it would take to gather that new piece of information. Put together, this decision process follows a random walk or diffusion process where the decision maker sets a threshold level of accuracy (balance of evidence) and gathers new information until that balance is reached[5]. The optimal balance of evidence may change over time[6], but this accumulation-to-threshold strategy will maximize a decision maker's subjective expected utility across a wide range of choice environments.

Consider a choice scenario where a decision maker has two options they could select. They make their decision by gathering new pieces of information, which they use to sequentially update their beliefs about a stimulus. In order to achieve a particular level of accuracy while minimizing response times, the optimal strategy is to track the balance of support s for option A versus option B, and stop when this balance exceeds a threshold value θ. In the case where time is costly (spending time results in negative utility) or where there is a mix of difficulty in a sequence of choices, this threshold will decrease over the course of each decision[6]. Each new piece of information can increase or decrease the balance of support. Thus, the rational decision maker gathers new information piece by piece until either $s \geq \theta$ or $s \leq -\theta$[5,7]. Here, we derive the balance of support at which a rational decision maker will stop and choose, showing that the resulting balance of support will follow a polarized distribution and create outlying extremists.

Suppose that new information is sampled from a true underlying distribution $f(x)$ that defines a distribution of evidence values it could take on $-\infty < x < \infty$ such that $\int_{-\infty}^{\infty} f(x) = 1$. The function $F(x)$ is the cumulative distribution function, determining the probability that the value of a new piece of information x_1 is less than or equal to x.

[1]A similar claim can be said of multialternative choice, so our proof applies there as well[4].

For now, we assume that all decision makers start in an unbiased state represented by the random variable $s(t)$, such that $s(0) = 0$.[2] Our goal is to derive the distribution of evidence states, specified by a probability density $g(x)$ over evidence levels x, that will result from a population of decision makers starting at evidence state $s_0 = 0$ and sampling new information from $f(x)$ until they reach state $\pm\theta$. We will designate the distribution of states of decision makers who finish at time t as $g_t(x)$, and the states of decision makers who have not finished before time t as a random variable $s(t)$.

The likelihood that a decision maker will stop after sampling a single piece of information x_1, drawn randomly as $x_1 \sim f(x)$, is simply the probability of sampling a piece of information that moves the decision maker past one of the thresholds, $Pr(t = 1) = F(-\theta) + (1 - F(\theta))$. The distribution of evidence among those decision makers who have enough support to stop after this first piece of information, $g_1(x)$, will be

$$g_1(x) = f(x|x \leq -\theta) + f(x|x \geq \theta) \tag{1}$$

Meanwhile, those decision makers who do not stop after a single piece of evidence will be distributed between $-\theta$ and θ. The "unfinished" states at time point 1 will be s_1, distributed according to a truncated distribution of $f(x)$:

$$Pr(s_1 = x) \sim \frac{f(x| - \theta < x < \theta)}{1 - Pr(t = 1)} \tag{2}$$

Those decision makers who have not made a selection yet will have to sample another piece of information from the source distribution $f(x)$, giving $s_1 + x_2$ where x_2 is a random variable drawn from $f(x)$. Some of these decision makers will then be able to stop and make a choice, if they have passed the threshold. Thus, the distribution of decision makers who have enough support to stop after two pieces of information will be

$$g_2(x) = Pr_x(x = s_1 + x_2) \cdot [Pr(x \leq -\theta) + Pr(x \geq \theta)] \tag{3}$$

where $x_2 \sim f(x)$. This procedure of adding a random variable from $f(x)$ to the existing distribution of states s_n, calculating the distribution below $-\theta$ / above θ, and truncating the remaining distribution of states can be carried out *ad infinitum* to get the distribution over evidence states that finish at each time point n.

$$g_n(x) = Pr_x(x = s_{n-1} + x_n) \cdot [Pr(x \leq -\theta) + Pr(x \geq \theta)] \tag{4}$$

Put together, this specifies a series of functions $g_1(x), g_2(x), ..., g_n(x)$ that specify the distribution of evidence among decision makers that stop after the first, second, ..., n^{th} piece of information. Importantly, the probability of stopping on a particular step t, given that the participant has not stopped yet, is given by the integral of $g_n(x)$:

$$Pr(t = n|t > [n-1]) = \int_{x=-\infty}^{\infty} g_n(x)dx. \tag{5}$$

Therefore, we can calculate the cumulative probability of stopping at or before step t as $G(t) = Pr(t = n|t > [n-1]) \cdot \big(1 - G(t-1)\big)$, noting that $G(1) = \int g_1(x)dx$ and calculating subsequent values iteratively. Because $s(t)$ follows a Wiener process, there are well-established methods to determine $g(t)$ and $G(t)$[8,9]. The overall distribution of evidence across decision makers at the conclusion of the decision process $S_t(x)$ can be then computed as

$$S_t(x) = \sum_{t=1}^{\infty} g_t(x) \cdot \big(1 - G(t)\big) \tag{6}$$

We could use $S_t(x)$ as the final distribution of evidence among decision makers who have finished deciding. However, it seems to make more sense for a decision maker's opinion to be determined by the *average* of all of the pieces of information they have collected[10], rather than as the *sum* of these pieces of information. (The summing rule makes the somewhat unreasonable prediction that there will be no decision makers whose beliefs lie between $-\theta$ and θ, and that participants' views can be more extreme than any pieces of information they could consider.) The distribution of average evidence for decision makers arriving at a choice at time t is computed by simply mapping $g_t(x) \rightarrow g_t\left(\frac{x}{t}\right)$, stretching or shrinking the x-scale by the number of pieces of information that had been drawn at time t.

[2] Variations on this assumption are explored in the model simulation section.

Polarization analyses

There were four main analyses carried out in the main text, whose results are presented in Table 1 and the Results. For the variance ratio analyses, we compared the variance of the observed information collected by participants against the expected variance of information that participants would have collected from a purely random sample (i.e., whose size did not depend on the stopping rule during choice). For Studies 1, 6, and 7, this was trivial: the exact stimulus values that could be generated on each trial were known, so we could use the objective variance on each trial along with the number of samples drawn on each trial to calculated the expected variance of the mean of the sample if it were representative. For these studies, the stimuli either generated multinomial random variables or normal random variables on each draw. Thus, we could calculate the expected variance of the mean of the multinomial / normal random variable for a given number of samples (given by the number the participant drew). This was calculated for every trial of every study, giving us a distribution of expected variance of the stimuli against which we could compare the observed variance of participants' samples.

As we might expect, the variance of observed and expected samples were generally quite close for these studies (Variance ratio in the first column of main text Table 1: Dots, DFE, and FGT).

Variance for the eye tracking studies was computed by taking the expected mean and variance for each individual fixation. It is the average of the squared differences between the expected value μ and the n attribute values a:

$$\sigma^2 = \frac{1}{n}\sum_{i}^{n}(a_i - \mu)^2$$

These were then used to calculated the expected value and variance of the mean for a trial, which was simply the sum of the the multinomial random variables describing the values we would expect them to sample on each fixation divided by the number of fixations. This allowed us to compute the expected variance of the mean for Studies 2-5 (Currency, Foood, DFD, and Posters), against which we compared the observed average difference across fixations.

In contrast to the Dots, DFE, and FGT studies, the eye tracking studies tended to show particularly extreme patterns of variance. This seems to be due to a tendency toward autocorrelation in fixations – participants will look at the same option for multiple fixations (each typically around 250 ms), and tend to look at the option they are about to select for several fixations especially leading up to choice. This is referred to as the *gaze cascade*[11], where the entropy of fixations reduces dramatically, thus distorting the information they consider relative to an unbiased sampling distribution. This gaze pattern provides evidence for the use of the relative evidence (rational / optimal) strategies that underlie the pattern of polarization observed in the data[12].

Simulating Polarization

Although they validate our predictions related to both polarization and extremism, it is useful to generate predictions and characterize choices beyond the scope of empirical studies we present in the paper. We do so here with simulations, which permit us to explore the shape of distributions that manifest across many thousands of trials as a function of important parameters of the model.

The three most important parameters of a random walk / diffusion model related to distribution of evidence are the direction / rate of evidence accumulation (drift), the stopping rule (threshold), and the starting point bias (prior bias)[13]. We manipulate each of these parameters in our simulations in order to explore their effect on polarization and extremism. Drift describes the information that participants gather, both in terms of what the stimulus provides and in terms of what the participants seek out themselves. It denotes the strength of an average piece of information that a participant will sample during the information accumulation process. Bias in the drift rate, usually modeled as the addition of a fixed constant[14], would reflect a decision maker's tendency to intentionally or unintentionally sample information that agrees with a particular position, including a confirmation bias in information seeking.

The second main parameter in our simulations, threshold, governs how much information a decision maker wishes to gather before they are certain enough of their position to make a choice. As we suggested in the main text, lower thresholds result in a decision maker basing their choices on fewer pieces of information, because they are willing to stop sooner. Classic manipulations of thresholds include incentives encouraging fast (low threshold) or accurate (high threshold) responses, such as time pressure or harsh punishments for incorrect answers[2,15]. As we suggested in the main text, lower thresholds tend to result in a greater incidence of extremism. The reason for this will become apparent in the simulations.

The final parameter that we manipulate in our simulations is one that has not been discussed at length thus far. Start point bias describes the beliefs or preferences that a decision maker has before they gather any information at all, meaning that a decision maker needs some reason to favor one response over the other in absence of any new information. This could occur naturally in political spheres, where a new policy can have biases for or against it due to the nature of people's views on the issue or the way it is associated with political positions. However, start point bias tends to appear less frequently in experiments where all the stimuli are new and/or randomly generated. The main way to manipulate start point biases is to

incentivize responses on one side or another (for example, paying participants more if they respond "right" than "left"), or where participants get some predecision cue as to which option will be correct[16].

Simulation results

The results of changing drift (accumulation bias), threshold, and start point (prior bias) are shown in Figure S1. We examine three main properties of the distributions: the proportion of responses favoring the right (+) or left (-) option, how widely spread decision makers are across the evidence space (width of the distributions) and the prevalence of extremists, which appear as "bumps" in the distributions far away from zero. Consider the top left panel, for example. There is an even distribution of responses favoring right and left alternatives (resulting from now drift bias), but responses are quite widely spread (high polarization) and there are substantial bumps in the distribution at around ± 0.5 (indicating a high prevalence of extremists).

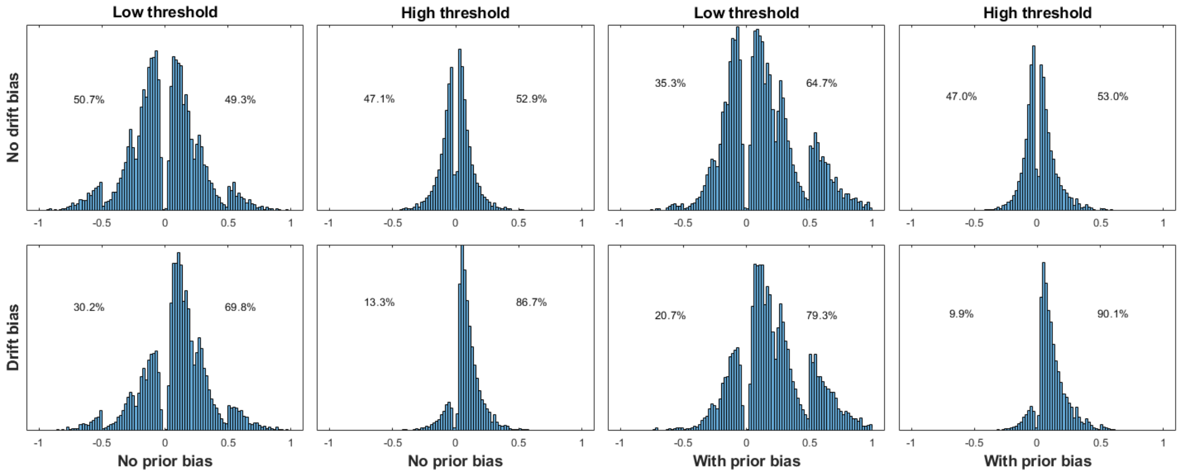

Figure S1. Distribution of evidence among populations of decision makers with varying drifts (top vs bottom), thresholds (colums 1/3 vs 2/4), and starting point biases (left vs right). Low thresholds tend to promote both polarization and extremism, while start point biases have a large effect on extremism in particular.

The top row of Figure S1 corresponds to cases where there is no drift bias and the information that participants gather or seek out does not systematically favor one option or the other, while the bottom row shows cases where participants systematically consider more information for the response option on the right (positive drift). From the simulations, it is clear that changes in drift actually have relatively minimal effects on the shape of the positive or negative sides of the distribution; instead, it simply determines what proportion of responses favor the left or the right option (choice proportions are provided on either side of each panel). This indicates that extremism is probably not the result of information search biases, because manipulating drift does not inherently increase the proportion of decision makers who occupy extreme positions – the bumps do not increase in size relative to the overall density of their respective side of the evidence distribution.

The first and third columns of Figure S1 correspond to cases where decision makers adopt a low threshold for their decisions, while the second and fourth columns correspond to cases where decision makers adopt a high / strict threshold. This appears to have two effects: first, the overall distribution is more tightly distributed near zero, indicating a lower degree of polarization among decision makers. This should not be too surprising: the mean of a larger number of values (pieces of information) will have less variability than the mean of a small number of values (pieces of information), meaning that participants will wind up closer to the true average distribution of information in the environment as they increase their thresholds. The second effect of thresholds is to moderate the prevalence of extremists. As illustrated in the figure, there are larger bumps (more extremists) when thresholds are low than when they are high. This occurs because the first and second pieces of information that participants gather are less likely to terminate the decision making process, because they are unlikely to push a decision maker far enough in one direction to pass a high threshold. As a result, the probability of stopping with very little information ($Pr(t = 1) = F(-\theta) + (1 - F(\theta))$ in the proof above) is much lower when θ is large. Thus, one potential way to reduce the prevalence of both polarization and extremism is to induce higher thresholds in decision makers, through interventions encouraging accuracy and thorough consideration of evidence and discouraging fast and inconsiderate choice strategies. However, the overall shape of the distribution of information in the population will never truly reflect the input distribution. There is always a gap between those favoring the option on the left and the option on the right, meaning that polarization is an unavoidable consequence of decision making when agents implement an optimal balance-of-evidence stopping rule.

Finally, the left panels of Figure S1 correspond to situations with no starting point bias, while the right panels correspond to situations with a starting point bias toward the right-hand (+) response. As shown, starting point bias has a quite substantial effect on the prevalence of extremists – whereas they constitute a relatively small proportion of decision makers even in the no bias conditions, the proportion of extremists nearly triples when start point bias goes from 0 (left) to 0.4 (right). This occurs because decision makers who already favor positive responses require very little information to reach the positive boundary – many decision makers will stop after receiving a single piece of information favoring the option that they are already biased toward. As a result, the prior distribution of beliefs among decision makers will have a drastic effect on extremism – a wide start point distribution will result in many extremists on both sides, while a narrow start point distribution will reduce the prevalence of extremists on both sides.

In one sense, this is quite intuitive: when people start with more extreme beliefs already, they make up their minds very quickly and are likely to to continue to hold extreme views. Our simulations additionally suggest that the decision making process will accentuate this extremism. Those that start with extreme views are likely to stop immediately after gathering information that makes them more extreme, and less likely to stop after sampling information that makes them less extreme. The decision making process therefore takes starting point biases and increases the bias in the population: the more decisions we make on related topics (where starting points could be expected to carry from one decision to the next), the more extreme our views become.

In many ways, this resembles interrogation and indoctrination strategies: have the subject admit to (decide in favor of) a small positive view, and then build on this small positive bias to make their views more extreme by iteratively offering them the chance to express (decide in favor of) more and more extreme views[17]. The similarities between indoctrination and polarization by sequential decisions are chilling, and our simulations suggest that they probably draw on a common mechanism: starting point bias. Further work on polarization and extremism should therefore consider the interaction between prior biases and decision making a central mechanism, worthy of deep consideration and exploration.

Verifying choice predictions with existing data

To examine whether polarization and extremism appeared in empirical work on decision making, we analyzed the results of seven different publicly-available decision making data sets. The tasks in these data sets varied in the type of stimuli and decision domain (preferential, inferential), but they had three important characteristics in common that made it possible to examine polarization and extremism: (1) the main goal of participants in the experiments was to make a series of decisions between binary choice options; (2) the true information provided by the stimulus was known, and tracked by the experimenters; and (3) each experiment recorded the final (average) set of information that participants had gathered on each trial. The reason behind the first criterion is that we are interested in the decision-making process and how it creates polarization. The other criteria are important because we need a benchmark against which to compare the empirical data. K-L divergence, and the plots shown in Figure S3, require an observed distribution (meaning the original experimenters had to record the information participants saw) and a reference / true distribution (meaning the stimulus distribution had to be known).

We should emphasize that this was not meant to be an exhaustive analysis of all studies that could have met our criteria, but a convenience sample of all the freely available studies on OSF we could find that included all the information we needed to carry out the analyses. A more detailed summary of the data from each study is provided below. We refer readers to the original studies / OSF pages for details on the samples, but we provide a short description of the seven experiments we analyzed here:

Study 1 - Dots[18] In the dots study, participants were presented with a series of dots that appeared on the screen and asked to decide whether the dots were being pulled from a pool of dots that was 2/3 green and 1/3 pink or 2/3 pink and 1/3 green. From trial to trial, the proportion of dots that were actually pink or green (strength) was manipulated, as well as the rate at which these dots were presented (weight). Note that this presented a confound for threshold analyses, as thresholds in the original study were found to increase with higher values of strength and weight manipulations[18], creating a violation of selective influence that would artificially increase the correlation between threshold estimates and the amount of evidence that participants gathered. This study included a total of 29 participants who each completed $\sim$200 trials of the task.

Study 2 - Currency[19] In this study, participants saw three currencies for each of two response options. Their task was to pick the set of three currencies that had the higher overall value (i.e., would yield the greatest total value sum). The currencies varied in type, including yen, pounds, and Q (made-up currency), and participants were given the exchange rates of each of these currencies to US dollars. Participants' gaze was tracked during the study so as to examine which of the outcomes (which of the six currencies) they were considering on each fixation. Positive and negative "sides" were randomly assigned such that the currencies on the top (bottom) conferred positive value while the currencies on the bottom (top) conferred negative value. This allowed us to examine the overall value of the pair of options: more positive indicated that the top (bottom) had greater value, while more negative indicated that the bottom (top) had greater value. On half the trials, the currencies displayed during a trial were all different (all three of yen, Q, and pounds for both options), and on the other half, the currencies displayed on a trial were all the same. We collapse across conditions for the analyses performed in this paper because they are not relevant to

our research questions. Participants responded by pressing one key (up) to select the first option on the screen, or another key (down) to select the second option. This study included a total of 46 participants, who each completed 84 trials of the task.

Study 3 - Food[20] In the food study, participants saw two snack foods displayed on either side of the screen and were instructed to decide which one they preferred. Before the decision trials, participants rated each of the 50 snack foods that could be displayed as stimuli in the experiments on a 1-9 scale in terms of desirability. These desirability values were used as inputs for the analyses we used in this paper – for example, a food with desirability value of 4 vs one with a desirability value of 6 would have a value difference of 2. As in the currency study, fixations were tracked and recorded. To get sample values from the eye tracking data, the desirability values of each option were centered so that fixating on the option with desirability 4 would have a value of -1, and fixating on the option with desirability of 6 would have a value of +1. A "representative" sample in this study was generated by randomly drawing the rated value of one snack or the other (50-50 chance). A total of 41 participants participated in this study, and each participant completed 100 trials.

Study 4 - Lotteries[21] In the lotteries study, also referred to as a decisions from description study, participants were tasked with making decisions between two gambles. Each gamble had three equally likely outcomes, one each in the 10-30, 40-60, and 70-90 range. As in the currency study, values on the top / bottom were assigned positive / negative values so that positive outcomes for one option were contrasted against positive outcomes for the other option, providing a net expected value between choice options and allowing us to evaluate the variance of the values sampled on each fixation. A total of 54 participants completed 100 trials each of this study.

Study 5 - Posters[19] The posters study was similar to the food study, except that Likert scales were gathered after the choice task rather than before. A "representative" sample in this study was generated by randomly drawing the rated value of one poster or the other (50-50 chance). A total of 53 participants completed the task and had usable data, and each one completed 100 trials of the study.

Study 6 - Decisions from experience[22] In the decisions from experience study, participants were tasked with both learning about and choosing between two gambles. In contrast to the decisions from description study, participants had to learn about each of the gambles by sampling from each one, rather than viewing the probabilities directly. For example, they could experience a series of samples [$4, $0, $4, $4, $0, $4] from a gamble that was 75% chance of $4 and 25% chance of $0. The different choice problems varied in terms of the number, magnitude, and probability of the different outcomes that participants could receive from either choice option. Once participants indicated they were finished sampling, they chose one option or the other. The exact sequence of draws obtained by a participant in this task was recorded and used for our analyses – as in the other studies, one option was coded as negative and the other as positive in order to determine the expected and observed variance / mean of the samples that participants drew. A "representative" sample in this study was generated by drawing randomly between the available decks (50-50 chance) and generating outcomes according to their objective probabilities. A total of 104 participants completed the study, and each one completed 228 trials.

Study 7 - Flash gambling[23] In the flash gambling study, participants were tasked with determining which of two flashing patches of dots had a greater mean number of dots (inference) or which one they would prefer to take a random draw from, given that more dots meant a greater payoff (preference). One patch of dots had a constant number of dots (130), and the dots simply changed positions from frame to frame. For the other patch of dots, a random number was generated with a known mean (e.g., 140) and standard deviation (e.g., 20) on each frame, and the resulting number of dots was displayed. The difference between the certain and uncertain patches therefore followed a normal distribution, which was used to define a "representative" draw for our analyses. However, the patches were refreshed at 60 Hz, making it very difficult to stop the stimulus immediately after obtaining a streak of information favoring one option or another. As a result, this study showed rather weak polarization and extremism results (Tables S1 & S2). A total of 113 participants each completed 960 trials of the experiment.

Each of the existing data sets we analyzed came from an open data set hosted on the Open Science Framework (OSF). The dots data are provided at osf.io/ba5c7; the currency, food, poster, and lottery / DFD data are provided at osf.io/mvk95[24]; the decisions from experience / DFE data are provided at osf.io/ngc45; and the flash gambling task data (Study 1) are provided at osf.io/g7a49. These studies were chosen so that we knew the data were (1) coming from an incentivized decision making task, (2) tracked the information that participants considered during the decision process, and (3) had all of the necessary information to compute the expected mean and variance of evidence of a random sample for the stimuli on each trial. We summarize the essential characteristics of the methods from each study here, but more detailed descriptions and explanations can be found in the original papers.

Studies 1, 6, and 7 were experiments in which participants received information piece by piece, allowing us to directly compare the information they saw against the information we would expect based on a random draw from the stimulus. Studies 2-5 were eye tracking studies that compared the value of stimulus attributes that participants looked at (observed) against the expected value of the stimuli that participants would have sampled if they looked randomly at stimulus attributes for the same number of fixations. These three characteristics allowed us to examine how the true information generated by the stimulus compared to the information that participants had gathered at the end of the decision making process.

All of the analyses presented in this paper used a Bayesian method for estimation or hypothesis testing (model comparison) with uninformative priors on all parameters (wide normal for unrestricted range, gamma for positive-only, or uniform for range-restricted parameters) that allowed the data to supplant the priors in determining the results. Analyses were implemented in JAGS[25], using MCMC sampling to estimate the posterior distribution of the statistic of interest (variance ratio, correlation, etc.). For all analyses, we present the mean estimate and the 95% highest density interval (HDI) for each effect[26].

To test for polarization, we compared the true stimulus information (representative samples) against the information that participants actually collected in each of the data sets. The first analysis we carried out directly compared the distributions of observed samples collected by participants against the distribution of samples we would expect to obtain from a random, representative sample. This allowed us to say how much participants differed from the – typically unimodal – distributions of evidence that a true random sample would provide.

To test for extremism, we compared the variance of the samples that participants gathered against the variance of a representative sample. This provided us with a measure of how extreme participants' sampled information was, as a ratio between the views they formed and the true information in their environment. We present a formal description of each of these analyses next.

Polarization analyses

The analyses for polarization followed those presented in the main text, using the Kullback-Leibler divergence [KLD][27] to measure the difference between the expected unimodal distribution of evidence and the evidence that participants actually collected during the experiment. The K-L divergence was approximated by calculating the probability density of the expected and observed distributions of evidence for a large number of points – 1001 evenly-spaced points from the minimum to the maximum of the observed data points or of the possible outcomes (for bounded scales like in the Dots study). This was used to approximate the integral

$$KLD[p(y)||p(x)] = \int_i^N p(\mathbf{y}_i)\log\left(\frac{p(\mathbf{y}_i)}{p(\mathbf{x}_i)}\right)$$

where y is the observed distribution and x is the expected distribution, with their probability densities at location i given by $p(x_i)$ and $p(y_i)$.

The density of the observed distribution was calculated by passing an optimal kernel density estimator over the observed data (the default values of kernel bandwidth in MATLAB's ksdensity being optimal for normal distributions[28]) to calculate a probability density at each of the points i.

However, this only gives a point estimate of the degree of polarization in the data. To construct a 95% credible interval around this estimate, we generated 10,000 simulated data sets from the expected distributions. This was carried out by first computing the true probability density function for the expected distribution (needed for the KLD calculation anyway), then using the cumulative probability density to perform inverse transform sampling[29] to sample N random draws from the representative / expected distribution, where N was the number of data points in the observed data.

For each of the 10,000 simulated data sets, which matched the observed data in size, we then calculate the KLD between the simulated data and the true expected sampling distribution using the same kernel density approximation method. This gave us a sample of 10,000 KLD values that described the distribution of KLDs we would obtain if the data were generated from the expected distribution. Once this was computed, we could compare the KLD values between the observed data and the 10,000 simulated data sets. This allowed us to construct the mean difference and a 95% credible interval around the difference between the observed KLD and the expected KLD (from the simulated data) from a representative distribution. The results of this analysis for each study are reported in Table S1 below.

Extremist analyses

To examine the degree of extremeness in participants' views, we compared the expected variance of the stimulus information (if a participant drew n samples at random, what would we expect the variance to be?) against the true variance of the information collected by participants (what was the variance of the n samples they actually collected?). We refer to this as the *variance ratio* analysis, specifying the ratio of observed variance to expected variance. The variance ratio for each experiment is provided in the first column of Table S2: Variance ratios greater than 1 indicate that participants collected more extreme information/were more polarized than we would expect purely by chance, while variance ratios less than 1 indicate that a participant collected less extreme information/were less polarized than expected.

In addition to the variance ratio analyses, we also calculated the extremeness of each trial and examined how individual differences in extremism were related to other patterns of behavior on the task. Specifically, we looked at correlations between the amount information participants gathered, the extremeness of the information they collected on each trial, and the rate

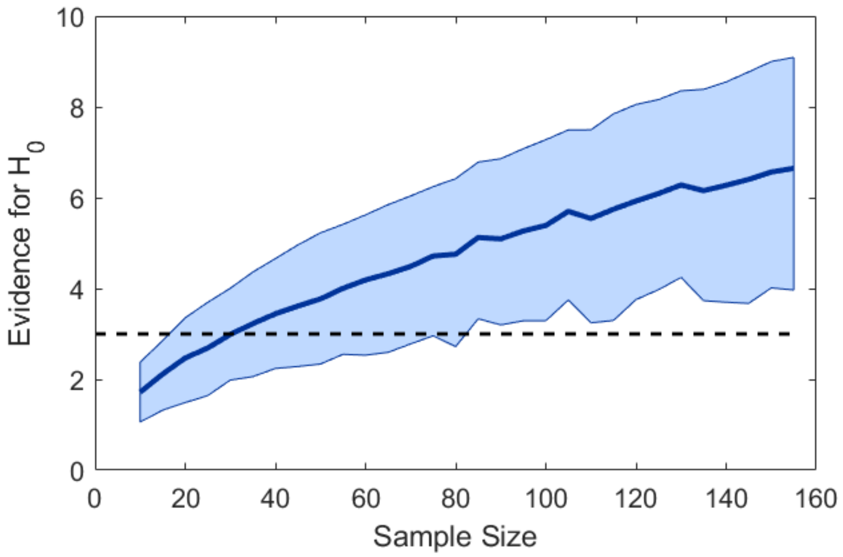

Figure S2. Relationship between sample size (x) and the average Bayes factor in favor of the null hypothesis (y). The average across 10,000 simulates is shown as the dark blue line, while the shaded region indicates the 20th and 80th percentiles of Bayes factors for each simulated sample size.

at which they made their decisions (response time). The degree of extremeness of the evidence participants gathered on a particular trial was defined as the absolute value of the average evidence they collected on each trial minus the true mean of the stimulus on that trial. This extremeness was correlated with the number of samples gathered on each trial (Information, Column 2 of Table S2) and the response time on each trial (Speed, Column 3 of Table S2).

Each of these correlation analyses were performed using Bayesian methods. The values for amount of information and response times were standardized so that the resulting correlations would be on a $[-1,1]$ scale. The prior for the correlation between variables was a uniform distribution $U(-1,1)$, and they were estimated using JAGS[25]. The posterior was generated using 4 chains of 5000 samples, with 500 burn-in steps in each chain and starting points generated randomly from the prior. These same settings were used to estimate the polarization effects as well, except that the variance ratio had a wide uniform prior $U(0,1000)$ and the extremity had a wide normal prior $N(0,1000)$. This allowed us to approximate the posterior distribution of each of the effects – the means and and 95% Highest Density Intervals (95% HDIs) of the effects are reported in Table S2.

Sample size rationale

The existing data sets had already been collected, and therefore there is not much to be done about their size or composition. Despite this, they are quite large and thorough: put together, there were a total of 440 participants and over 150,000 trials across these data sets. They spanned inferential and preferential choice, eye tracking and sampling paradigms for gathering information, and both description and experience-based types of information. This wide-ranging set of studies suggests that the phenomena we observe are pervasive, and in particular that polarization and the presence of extremists are consequences of adopting relative-evidence decision strategies regardless of the particular domain of choice.

For the new studies presented in the main text, we aimed to gather sufficient data to be able to relate the individual difference measures we collected (Dogmatism, Personal Need for Closure) to individual differences in sampling behavior such as the number of clicks of degree of polarization. We determined that we would like to be able to conclude in favor of the null hypothesis (no relationship between measures) at least 80% of the time when the null hypothesis was in fact true, an inference that is made possible by the use of Bayesian statistics[30]. To estimate how many participants would be needed, we created 10,000 artificial data sets at differing sample sizes, shown in Figure S2, where the relationship between two variables was zero (i.e., the null hypothesis was true). For each artificial data set, we computed the Savage-Dickey Bayes factor[31] quantifying the degree of support for or against the null hypothesis. Bayes factors greater than 3 (less than 1/3) indicate solid support for (against) the null. We determined that 85 participants was sufficient to ensure that we would support the null with a Bayes factor of 3 or more in at least 80% of our analyses.

In order to obtain a more representative sample of U.S. participants (which we focused on because of the nature of the political topics) than we might expect from a university undergraduate sample, we recruited online through Prolific Academic.

Table S1

Study	Expected KLD	Observed KLD	Difference	95% HDI
Dots	3.85	4.09	0.25	[-0.75, 1.27]
Currency	0.02	0.74	0.73	[0.72, 0,73]
Food	0.03	0.31	0.28	[0.27, 0.29]
DFD	0.0069	0.4	0.39	[0.38, 0.39]
Posters	0.028	1.28	1.25	[1.24, 1.26]
DFE	0.018	2.56	2.54	[2.53, 2.54]
FGT	0.0004	0.0086	0.0082	[0.0081, 0.0084]

Table S2. Table of mean effects [95% HDIs] related to extremism for each of the seven studies. The variance ratio is the relative variance of the observed versus expected samples (greater than 1 indicates polarization). For extremists, we examined the relationship between the extremeness of evidence on a particular trial and the amount of information gathered on that trial (Information) and the response times on those trials (Speed).

	Extremism	Relation to other measures	
Study	Variance ratio	Information	Speed
Dots	1.21 [1.16, 1.26]	-.39 [-.42, -.36]	-.41 [-.44, -.39]
Currency	7.24 [6.86, 7.69]	-.11 [-.15, -.07]	-.07 [-.11, -.03]
Food	3.21 [3.08, 3.36]	-.04 [-.07, -.01]	-.14 [-.17, -.10]
DFD	3.28 [3.09, 3.51]	-.10 [-.15, -.05]	-.14 [-.19, -.10]
Posters	8.51 [8.09, 9.98]	-.04 [-.08, -.00]	-.17 [-.20, -.13]
DFE	2.13 [2.10, 2.17]	-.19 [-.20, -.17]	-.23 [-.24, -.21]
FGT	0.78 [0.77, 0.79]	-.25 [-.26, -.24]	-.55 [-.56, -.55]

Results

The analysis for polarization compared the KLD of the observed data relative to the true probability density (observed KLD) against the results of 10,000 matched-N random samples from the true distribution relative to the true probability density (expected KLD). This allowed us to estimate a 95% highest density interval on the differences between the observed polarization and the degree of polarization we would expect to obtain just by chance. The results of this analysis are shown in Table S1 for each study we re-analyzed.

Out of the seven studies, six showed a difference in KLD between the observed data and the random samples from the true distribution. For the Currency, Food, DFD, Posters, and DFE studies, the KLD was several times greater than we would expect purely by chance, resulting in stark differences between the observed KLD and the distribution of expected KLDs. This suggests a large degree of polarization in the information collected by participants in these studies, who gathered information in a way that biased them away from the center of the distribution / more moderate information. For the Dots study, the observed KLD was larger than the expected KLD, but the random samples from the true distribution had sufficient variability that the observed KLD was not large enough to constitute a credible difference. The FGT study also showed a credible KLD difference, but this may actually run in the opposite direction to the polarization effect observed in the other studies (see Figure S3) and indicate that the difference between distributions (which was quite small anyway) was driven by more observed samples near 0 than would be expected from a representative sample.

In addition to the polarization analyses, we examined the extremeness of the information that participants collected in each study, defined as the average value of all the pieces of information they collected during a trial. Aggregate estimates of extremeness were created by combining these trial-level outcomes into individual differences (by aggregating within participants) and into an overall extremeness score for each of the experiments (by aggregating across participants). The variance of the study-aggregated samples was compared to the expected variance from a representative sample, giving the first *variance ratio* column of Table S2. The individual-level extremism measures (aggregated only within subjects) were then correlated with important outcomes like how many pieces of information each person gathered (Information) and how quickly they made their choices (Speed). These linear correlations are presented in the second and third columns of Table S2, respectively.

In six of the seven studies, we found that the variance in the information gathered by participants was greater than would be expected given a representative sample from the distribution (Figure S3). The only study that showed a pattern of results that was inconsistent with a pattern of polarization was the Flash Gambling Task (FGT) study. Because information arrived rapidly in this study (the screen updated at 20 Hz), participants were likely unable to consider every single piece of information.

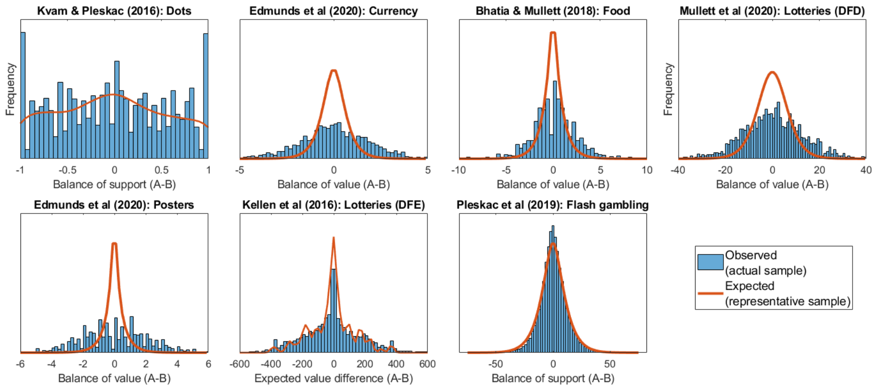

Figure S3. Distribution of observed information gathered by participants (blue histograms) compared to the expected distribution of information that a participant *should* have, given representative draws from the true distributions.

Furthermore, even if participants had made up their minds after gathering an extreme piece of information, information arrived continuously until they made their response. If the intervening period were around 200-300 ms[32], then they would have gathered at least 4-6 additional samples of evidence, diluting the extremity of the pool of information they appear to have considered before the end of the trial. This would naturally wash out any effects of the participants' stopping rules and negate the typical extremism effect that we observed in other studies.

The remaining studies showed a robust and large extremism effect, with the estimated variance of the samples participants collected ranging from 121% to 851% of the variance we would expect from random sampling. To illustrate this effect visually, the patterns of polarization in these studies are also shown in Figure S3. The expected distribution of information that we would anticipate from a representative sample is shown in orange, while the observed distribution of information collected by participants is shown as a blue histogram. Clearly, the blue histograms show a more widely dispersed pattern of information than the expected distributions (orange lines).

Put together with the polarization (Table S1) and extremism (Table S2) analyses, it is clear that the decision strategies participants implemented in these studies – with the possible exception of the FGT study – created a bias in the information they collected. These results constitute strong evidence for the effect of decision goals on polarization, suggesting that polarization and extremism can arise without motivated bias. Instead, polarization and extremism arise as a function of the information decision makers collect; and crucially, occurs even on simple perceptual tasks.

Extremists in the distribution

Although the presence of polarization is an important implication of widespread use of optimal choice strategies, it is not the only interesting phenomenon that arises as a consequence of implementing these strategies to make decisions. Among populations of decision makers, there will naturally be variation in the value that people place on making the correct (accurate, informed) decision versus making a timely decision. This trade-off between speed and accuracy is reflected in the setting of the threshold in dynamic models of decision making[2, 15]. As illustrated in the proof and simulations, some decision makers executing optimal strategies favoring speed will appear as "lumps" at the poles of the information distribution. These individuals tend to be decision makers who make up their minds after seeing only a few pieces of extreme information, emphasizing speedy sampling over thorough sampling when making decisions. They tend to be both polarized (have no views near the center of the distribution, high individual-level KLD) and adopt extreme views (high variance in collected information). For simplicity, however, we refer to them as *extremists*, as they tend to appear at the tails of the distributions of information and possess distorted views of the information that is in their environments. Extremism appears when a decision maker who favors speedy decisions (low thresholds) encounters a small set or even a single piece of extreme information. This piece of information allows them to immediately cross their threshold and favor $A \succ B$ or $B \succ A$. They can therefore stop gathering new information, meaning that the extreme view provided by the lone piece of extreme information is never combined or tempered with conflicting evidence.

Evaluating our first measure of extremism, we found the averages of smaller samples tended to be more volatile, and thus the extremists were likely to have collected less information than their peers (summarized in the Information column in Table S2). In all seven studies, this is evidenced by a credible (95% HDI excluding zero; significant, in classical statistical terms) negative correlation between the number of pieces of information and the overall extremity of that information. Similarly, our second measure found that in all seven studies, there was a negative relationship between response time and information

extremity, suggesting that participants with extreme views tended to make faster decisions during the experiments (Speed column in Table S2).

Another way to understand extremism is to explore underlying individual differences in the cognitive mechanisms supporting choice, which might offer insights into what made these particular individuals become extremists. We suggest that low thresholds, indicating a preference for speedy as opposed to well-informed decisions, is more common among extremists (Figure S1). To investigate this, we applied a simple diffusion model[13] to predict choices and response times in terms of the strength of information favoring one option over another (drift), priority for speed or accuracy (threshold), and the length of time spent on processes unrelated to choice such as encoding stimuli and making a response (non-decision time). For each trial of each study, the drift rate was fixed based on the true average difference in value between stimuli. Threshold and non-decision time were permitted to vary across participants (none of the studies manipulated speed / accuracy instructions), so that each person was assigned a unique value for their threshold. This allowed us to compare the average extremeness of an individual participant's responses (extremism) against a rough estimate of the threshold they used to make choices. This analysis was carried out for the five studies it was possible to apply the diffusion model[3].

This disentangled performance for each participant on each task into the strength of information provided by the stimulus (drift), the amount of time on each trial devoted to non-decision processes (non-decision time), and the amount of information participants wanted to collect before deciding (threshold). Because the first two measures are primarily related to the stimulus and to participants' motor abilities, we looked only at the threshold parameter. In four of the five studies, our analysis of individual differences in decision thresholds showed that there was a credible (significant) negative correlation between extremism and thresholds estimated for a diffusion model: Food ($b = -.04$, 95% HDI = $[-.05, -.03]$), DFD ($b = -.08$, 95% HDI = $[-.11, -.06]$), Posters ($b = -.06$, 95% HDI = $[-.08, -.04]$), and FGT ($b = -.04$, 95% HDI = $[-.05, -.03]$). While not overwhelming evidence, these results do suggest that decision makers with more extreme views tended to favor fast decisions over well-informed ones. We explore potential individual differences that may underlie this extremism effect in the new study, described in the following sections.

Alternative solutions to polarization

Although the estimation manipulation is the main approach we used to try to reduce polarization and extremism in information sampling, there are other possible approaches that may or may not work. One seemingly obvious strategy to reduce polarization in binary choices like those we present here, would be to simply offer more choices. The authors ourselves were hopeful that multi-alternative choice would curb polarization and extremism, thinking that polarization was an issue of having two opposing options and that adding a third could eliminate or reduce it. If true, the addition of another viable alternative to reduce polarization could provide justification for eliminating two-party systems (resulting from first-past-the-post) or for ranked-choice voting protocols as interventions.

Unfortunately, this was not the case – a similar pattern of polarization and extremism occurs beyond binary choice as well. Optimal choice strategies for these situations are provided in work by[4,33,34]: essentially, a decision maker's beliefs in optimal trinary choice can be described by a location on a triangle, and by a multidimensional simplex when there are 4+ options[4]. To make a decision, the decision maker starts on the interior of the triangle (simplex) and gathers information, changing their beliefs accordingly until they cross one of the edges of the triangle (sides of a simplex). The size of the triangle (simplex) is determined by the height of the thresholds. This means that extreme information is still more likely that moderate information to terminate the decision process, with the end result being the same as in binary choice.

This is illustrated in Figure S4: a fully symmetric distribution of input choice evidence creates three "pockets" of posterior evidence favoring each option, centered around those favoring A, those favoring B, and those favoring C. There are very few decision makers between A and B, B and C, and A and C, and yet fewer who have a near-even view of all three. In fact, across more than 100,000 simulations shown in Figure S4, we had no instances of decision makers who had a balanced or even nearly-balanced view of the three options (white area of the figure). More choice does not mean less polarization.

Absolute evidence

One final approach that may help reduce polarization and extremism is for people to represent support for different choice options separately, rather than as a balance between the options they have available. In accumulator models of decision making[35,36] , people are thought to implement an *absolute stopping rule*, where they gather support for each of the available choice options until the degree of support for one of the alternatives reaches a threshold. Despite making nearly identical predictions for the shape of response time distributions[37], accumulator models differ substantially in the pattern of information accumulation we observe. A simulation of these strategies is shown in Figure S5.

[3]We could not include the Dots study, as it contained a manipulation that artificially induced a relationship between the amount of information and speed of decision making[18], nor the DFE study, since it has a discrete sampling structure which meant that response times were not commensurate with the number of pieces of information drawn.

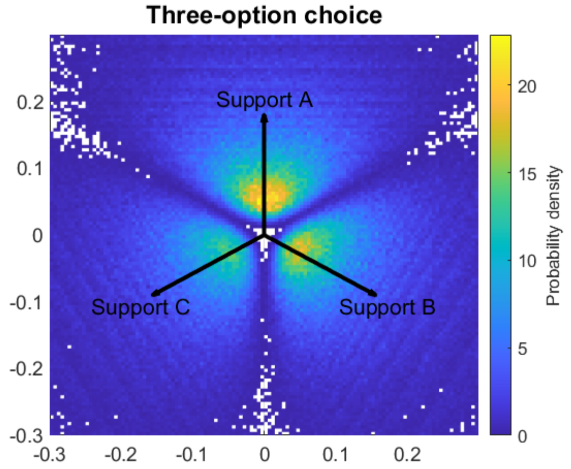

Figure S4. Distribution of beliefs (evidence) among a population of decision makers who have made a decision among three options A, B, and C.

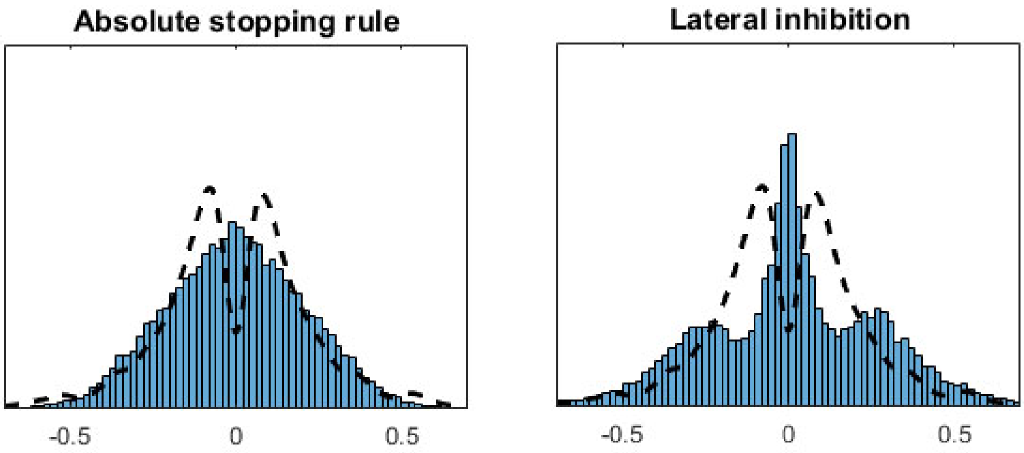

Figure S5. Simulated data from an accumulator model with no interaction between accumulators (left) and with lateral inhibition between them (right), compared against the distribution of information we would expect from a relative stopping rule (dotted black line).

As shown, an absolute stopping rule does have the potential to reduce polarization similar to estimation goals. By separating support into support for A, support for B, etc., information does not have to be extreme in order to trigger a choice. Instead, the support for each option is the sum of any small, moderate, or large increase in support; a decision maker does not have to be sensitive to the balance of support between options, just the overall degree of support for each one. As a result, extreme information loses some of its value in triggering a decision and the overall distribution of information that (simulated) participants collect reverts to a more representative one as shown in the left panel of Figure S5.

Whether representing support for different options separately will reduce polarization, however, depends on whether there are any interactions between support for different options. In many accumulator models of decision making, support for one option decreases the rate at which support for another option can increase, referred to as *lateral inhibition*[38,39]. These interactions between options can actually exacerbate the problem of polarization: if a person already strongly supports one option, they will not gather support for contrary options. As a result, people get "funneled" into strong support for one option or another as soon as they gather enough one-sided support for an option. As a result, they wind up in a tri-modal distribution of support: those who favor option A and will not consider information for option B, those who favor option B and do not consider information for option A, and those who maintain a relatively even balance of support for A and B throughout the accumulation process.

The result of many simulations of this strategy is shown in the right panel of Figure S5: instead of removing polarization, absolute stopping rules with interacting accumulators can exacerbate the problem of extremism. The effect of absolute stopping rules and the moderating effect of lateral inhibition are certainly worthy of investigation in future interventions seeking to reduce polarization and extremism.

Fixed sampling

Another way to reduce polarization is to simply force decision makers to take some number n pieces of information before stopping, as in fixed-sampling paradigms[40,41]. If the information that decision makers gather is independent and identically distributed (i.i.d.) across samples, as is typically assumed, decision makers will obtain a representative sample of the true information in the environment and thus polarization will not occur. However, this approach is paternalistic and thus potentially very difficult to implement[42,43], as it takes the autonomy of information collection away from decision makers[44]. In future studies, it would be interesting to test more benign versions of such interventions such as providing a large initial sample of information for decision makers to sort through before gathering additional samples.

References

1. Savage, L. J. *The Foundations of Statistics* (John Wiley & Sons, Inc, New York, NY, 1954).
2. Wickelgren, W. A. Speed-accuracy tradeoff and information processing dynamics. *Acta Psychol.* **41**, 67–85, DOI: 10.1016/0001-6918(77)90012-9 (1977).
3. Wald, A. & Wolfowitz, J. Bayes solutions of sequential decision problems. *Proc. Natl. Acad. Sci. United States Am.* **35**, 99–102 (1949).
4. Kvam, P. D. A geometric framework for modeling dynamic decisions among arbitrarily many alternatives. *J. Math. Psychol.* **91**, 14–37 (2019).
5. Bogacz, R., Brown, E., Moehlis, J., Holmes, P. & Cohen, J. D. The physics of optimal decision making: A formal analysis of models of performance in two-alternative forced-choice tasks. *Psychol. Rev.* **113**, 700–765, DOI: 10.1037/0033-295X.113.4.700 (2006).
6. Malhotra, G., Leslie, D. S., Ludwig, C. J. & Bogacz, R. Time-varying decision boundaries: insights from optimality analysis. *Psychon. bulletin & review* **25**, 971–996 (2018).
7. Edwards, W. Optimal strategies for seeking information: Models for statistics, choice reaction times, and human information processing. *J. Math. Psychol.* **2**, 312–329, DOI: 10.1016/0022-2496(65)90007-6 (1965).
8. Diederich, A. & Busemeyer, J. R. Simple matrix methods for analyzing diffusion models of choice probability, choice response time, and simple response time. *J. Math. Psychol.* **47**, 304–322, DOI: 10.1016/S0022-2496(03)00003-8 (2003).
9. Ratcliff, R. A theory of memory retrieval. *Psychol. Rev.* **85**, 59–108, DOI: 10.1037/0033-295X.85.2.59 (1978).
10. Turner, B. M., Gao, J., Koenig, S., Palfy, D. & McClelland, J. L. The dynamics of multimodal integration: The averaging diffusion model. *Psychon. Bull. & Rev.* **24**, 1819–1843 (2017).
11. Shimojo, S., Simion, C., Shimojo, E. & Scheier, C. Gaze bias both reflects and influences preference. *Nat. neuroscience* **6**, 1317–1322 (2003).

12. Mullett, T. L. & Stewart, N. Implications of visual attention phenomena for models of preferential choice. *Decision* (2016).

13. Ratcliff, R., Smith, P. L., Brown, S. D. & McKoon, G. Diffusion decision model: Current issues and history. *Trends Cogn. Sci.* **20**, 260–281 (2016).

14. Diederich, A. & Busemeyer, J. R. Modeling the effects of payoff on response bias in a perceptual discrimination task: Bound-change, drift-rate-change, or two-stage-processing hypothesis. *Percept. & Psychophys.* **68**, 194–207, DOI: 10.3758/BF03193669 (2006).

15. Heitz, R. P. The speed-accuracy tradeoff: history, physiology, methodology, and behavior. *Front. Neurosci.* **8**, 150 (2014).

16. Mulder, M. J., Wagenmakers, E.-J., Ratcliff, R., Boekel, W. & Forstmann, B. U. Bias in the brain: a diffusion model analysis of prior probability and potential payoff. *J. Neurosci.* **32**, 2335–2343 (2012).

17. Hinkle, L. E. & Wolff, H. G. Communist interrogation and indoctrination of enemies of the states: Analysis of methods used by the communist state police (a special report). *AMA Arch. Neurol. & Psychiatry* **76**, 115–174 (1956).

18. Kvam, P. D. & Pleskac, T. J. Strength and weight: The determinants of choice and confidence. *Cognition* **152**, 170–380, DOI: 10.1016/j.cognition.2016.04.008 (2016).

19. Edmunds, C. E., Bose, D., Camerer, C. F., Mullett, T. L. & Stewart, N. Experimental investigations of attention interaction in preferential choice. *https://osf.io/mvk95/* (2020).

20. Bhatia, S. & Mullett, T. L. Similarity and decision time in preferential choice. *Q. J. Exp. Psychol.* **71**, 1276–1280 (2018).

21. Mullett, T. L., Ravichandar, N. & Stewart, N. Attention and accumulation in risky choice. *https://osf.io/mvk95/* (in prep).

22. Kellen, D., Pachur, T. & Hertwig, R. How (in) variant are subjective representations of described and experienced risk and rewards? *Cognition* **157**, 126–138 (2016).

23. Pleskac, T. J., Yu, S., Hopwood, C. & Liu, T. Mechanisms of deliberation during preferential choice: Perspectives from computational modeling and individual differences. *Decision* **6**, 77–107 (2019).

24. Edmunds, C. E., Bose, D., Camerer, C. F., Mullett, T. L. & Stewart, N. Accumulation is late and brief in preferential choice. *PsyArXiv* DOI: 10.31234/osf.io/sa4zr (2020).

25. Plummer, M. JAGS: A program for analysis of Bayesian graphical models using Gibbs sampling. In *Proceedings of the 3rd International Workshop on Distributed Statistical Computing*, vol. 124, 10 (Vienna, Austria., 2003).

26. Kruschke, J. K. *Doing Bayesian Data Analysis: A Tutorial with R, JAGS, and STAN* (Academic Press, 2014).

27. Kullback, S. & Leibler, R. A. On information and sufficiency. *The annals mathematical statistics* **22**, 79–86 (1951).

28. Bowman, A. W. & Azzalini, A. *Applied smoothing techniques for data analysis: the kernel approach with S-Plus illustrations*, vol. 18 (OUP Oxford, 1997).

29. Vogel, C. R. *Computational methods for inverse problems* (SIAM, 2002).

30. Lee, M. D. & Wagenmakers, E.-J. *Bayesian Cognitive Modeling: A Practical Course* (Cambridge university press, 2014).

31. Wagenmakers, E. J., Lodewyckx, T., Kuriyal, H. & Grasman, R. Bayesian hypothesis testing for psychologists: A tutorial on the Savage-Dickey method. *Cogn. Psychol.* **60**, 158–189, DOI: 10.1016/j.cogpsych.2009.12.001 (2010).

32. Ratcliff, R. & McKoon, G. The diffusion decision model: Theory and data for two-choice decision tasks. *Neural Comput.* **20**, 873–922, DOI: 10.1162/neco.2008.12-06-420 (2008).

33. Link, S. W. & Heath, R. A. A sequential theory of psychological discrimination. *Psychometrika* **40**, 77–105, DOI: 10.1007/BF02291481 (1975).

34. Tajima, S., Drugowitsch, J., Patel, N. & Pouget, A. Optimal policy for multi-alternative decisions. *Nat. neuroscience* **22**, 1503–1511 (2019).

35. Smith, P. L. & Vickers, D. The accumulator model of two-choice discrimination. *J. Math. Psychol.* **32**, 135–168, DOI: 10.1016/0022-2496(88)90043-0 (1988).

36. Brown, S. D. & Heathcote, A. The simplest complete model of choice response time: Linear ballistic accumulation. *Cogn. Psychol.* **57**, 153–178, DOI: 10.1016/j.cogpsych.2007.12.002 (2008).

37. Donkin, C., Brown, S., Heathcote, A. & Wagenmakers, E.-J. Diffusion versus linear ballistic accumulation: Different models but the same conclusions about psychological processes? *Psychon. Bull. & Rev.* **18**, 61–69, DOI: 10.3758/s13423-010-0022-4 (2011).

38. Usher, M. & McClelland, J. L. The time course of perceptual choice: The leaky, competing accumulator model. *Psychol. Rev.* **108**, 550–592, DOI: 10.1037/0033-295X.108.3.550 (2001).

39. Usher, M. & McClelland, J. L. Loss aversion and inhibition in dynamical models of multialternative choice. *Psychol. Rev.* **111**, 757–769, DOI: 10.1037/0033-295X.111.3.757 (2004).

40. Busemeyer, J. R. Decision making under uncertainty: a comparison of simple scalability, fixed-sample, and sequential-sampling models. *J. Exp. Psychol. Learn. Mem. Cogn.* **11**, 538 (1985).

41. Fried, L. S. & Peterson, C. R. Information seeking: Optional versus fixed stopping. *J. Exp. Psychol.* **80**, 525 (1969).

42. Grüne-Yanoff, T. & Hertwig, R. Nudge versus boost: How coherent are policy and theory? *Minds Mach.* **26**, 149–183 (2016).

43. Hertwig, R. & Ryall, M. D. Nudge versus boost: Agency dynamics under libertarian paternalism. *The Econ. J.* **130**, 1384–1415 (2020).

44. Deci, E. L. & Ryan, R. M. Human autonomy. In *Efficacy, agency, and self-esteem*, 31–49 (Springer, 1995).
